# Prediction of disease progression and outcomes in multiple sclerosis with machine learning

**DOI:** 10.1038/s41598-020-78212-6

**Published:** 2020-12-03

**Authors:** Mauro F. Pinto, Hugo Oliveira, Sónia Batista, Luís Cruz, Mafalda Pinto, Inês Correia, Pedro Martins, César Teixeira

**Affiliations:** 1 Department of Informatics Engineering, Centre for Informatics and Systems of the University of Coimbra, University of Coimbra, Coimbra, Portugal; 2 Neurology Department of Centro Hospitalar e Universitário de Coimbra, Coimbra, Portugal; 3 Functional Unit of Neuroradiology, Medical Imaging Department, Centro Hospitalar e Universitário de Coimbra, Coimbra, Portugal

**Keywords:** Machine learning, Predictive medicine

## Abstract

Multiple Sclerosis is a chronic inflammatory disease, affecting the Central Nervous System and leading to irreversible neurological damage, such as long term functional impairment and disability. It has no cure and the symptoms vary widely, depending on the affected regions, amount of damage, and the ability to activate compensatory mechanisms, which constitutes a challenge to evaluate and predict its course. Additionally, relapsing-remitting patients can evolve its course into a secondary progressive, characterized by a slow progression of disability independent of relapses. With clinical information from Multiple Sclerosis patients, we developed a machine learning exploration framework concerning this disease evolution, more specifically to obtain three predictions: one on conversion to secondary progressive course and two on disease severity with rapid accumulation of disability, concerning the 6th and 10th years of progression. For the first case, the best results were obtained within two years: AUC=$0.86\pm 0.07$, sensitivity=$0.76\pm 0.14$ and specificity=$0.77\pm 0.05$; and for the second, the best results were obtained for the 6th year of progression, also within two years: AUC=$0.89\pm 0.03$, sensitivity=$0.84\pm 0.11$, and specificity=$0.81\pm 0.05$. The Expanded Disability Status Scale value, the majority of functional systems, affected functions during relapses, and age at onset were described as the most predictive features. These results demonstrate the possibility of predicting Multiple Sclerosis progression by using machine learning, which may help to understand this disease’s dynamics and thus, advise physicians on medication intake.

## Introduction

Multiple Sclerosis (MS) is an inflammatory immune-mediated disease of the Nervous Central System (CNS) that targets mainly the myelin. Besides being one of the most common neurological conditions, it is usually diagnosed in young adults from 20 to 45 years old and occurs more frequently in females. Thus, it has a significant social impact related to discrimination and stigma. Despite its heterogeneous and unforeseeable course whose impact varies between patients, this disease is initially characterized by relapses (periods of neurological problems that are reversible). Afterwards, a gradual neurological worsening is frequently observed^1,2^. Some of the most common symptoms are optic lesions and sensitivity decrease, pain, weakness and sensory loss in the arms/legs, fatigue, cognitive problems, and depression^3–5^.

Clinically isolated syndrome (CIS) refers to a first clinical CNS demyelinating event, which is compatible with the possible future development of multiple sclerosis (MS). If there is a subsequent series of events, defined by clinical relapses or by Magnetic Resonance Imaging (MRI) activity, a diagnosis of MS relapsing-remitting (RR) is established^6,7^. If a progressive accumulation of disability is observed from onset, the primary progressive (PP) course is defined while secondary progressive (SP) is characterized by a progressive accumulation of disability after an initial RR course instead. Thus, SP gradual development in patients who suffer from RR type may occur if the disease is not efficiently treated, where the neurological condition continues to worsen with or without remission periods^1,2,7,8^.

To study MS progression, the terms benign and malignant are also frequently used, which are not a standard pattern of classification but rather indicators of the disease severity over time. Benign MS is usually defined by few relapses and reduced/absence of disability after 20 years of evolution, while the malignant form is known for frequent disabling attacks along with incomplete recovery, resulting in fast progression of disability. The indicators criteria are not precise, as different experts tend to use different definitions through Expanded Disability Status Scale (EDSS)^1,2,7,8^ a scale proposed by Kurtzke^8^ to evaluate the neurological condition of an MS patient through time. The EDSS is based on the state of eight Functional Systems (FS): Pyramidal, Cerebellar, Brain Stem, Sensory, Bowel & Bladder, Visual, Cerebral or Mental, and Other or Miscellaneous. The higher its value is, the worse the neurological condition of the patient is, ranging from 0 (normal) to 10 (death by MS). Levels from 1.0 to 4.5 correspond to patients still having a high degree of ambulatory ability, while patients with levels from 5.0 to 9.5 have a severe loss of ambulatory ability. Thus, while some authors assume EDSS variations from 1 to 4 as benign, others may use a progression index that accounts for the disease duration or even the neurological functions evolution. The used benign definition will significantly affect the obtained results, as the proportion of benign/malignant patients has ranged from 6% benign to 64%^9^.

Besides predicting the disease progression, literature has attempted to describe its predictors, where a high relapse frequency and a high EDSS in the first years of disease evolution are some of the most reported regarding an unfavourable outcome^9–12^. In addition to clinical information, MRI^13,14^ and DNA^15^ have also been used. Female gender, race, family history of MS, brain parenchymal fraction, early-onset, optic neuritis, and sensory symptoms have been associated with a favourable course, while others as pyramidal involvement and brain T2 lesion volume were considered characteristic of a severe progression^9,10,13^. Moreover, Bengt Skoog et al.^16^ concluded that predictors in later stages of the course were more effective than the traditional onset predictors and that the number of potential ones could be reduced to a few.

However, some studies achieved contradictory findings concerning important predictors. For example, Reynders et al.^12^, suggested that the EDSS is associated with motor functions and thus, other characteristics are not usually assessed, such as mental and cognitive impact. It was also concluded that gender and age were not affecting the MS course but rather interacting with the clinical disease phenotype. Hawkins et al.^10^ concluded that although the EDSS may provide useful prognosis information, an apparent benign course can often become malignant.

Despite initial studies have been based on correlations and statistical tests^10,11,14^, machine learning models have been proved useful in several medical problems, where MS is not an exception. A machine learning methodology may provide a second opinion to a physician where a real-life situation can be applied, as the case of Zhao et al.^13^, Rodriguez et al.^15^, Seccia et al.^17^, Pellegrini et al.^18^ and Law et al.^19^. Machine learning has also been used in MS diagnosis^20,21^.

In this study, we present an exploratory framework with machine learning that aims at predicting MS progression, based on the clinical characteristics of the first five years of the disease. This progression prediction is based on two factors: the MS course (SP development/not development) and disease severity (related to the benign/malignant form) in patients initially diagnosed as RR. By developing machine learning models where progression information from consecutive years is accumulated, we obtain insight into how performance can change over time, which provides us with a certainty measure. Besides, it also enables the possibility to perform an exploratory study concerning the chronology of important predictors and its influence, which may allow a clinician to understand the patient’s disease progression and thus, support its decision concerning the best treatment strategy as several options are currently possible.

## Data & methods

**Figure 1:**
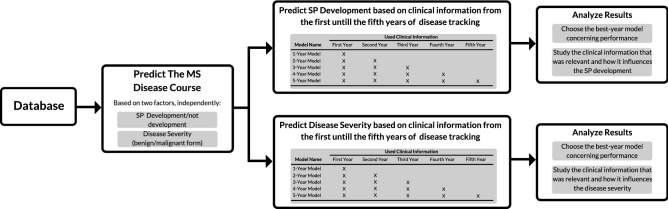
The followed methodology. Two independent problems were investigated: SP development and disease severity. For each prediction, one on SP development and two on disease severity, five year models were developed concerning the accumulation of clinical information on the first five years of progression. Then, the results were analyzed in terms of prediction performance and feature predictive power.

The followed methodology is illustrated in Fig. 1 and is described in this section. In short, we investigated two different problems: i) if a patient would develop its RR course into an SP one (SP development) and ii) if a patient’s disease progression would be, in the future (in the 6th and in the 10th year), severe with rapid accumulation of disability (disease severity). For each prediction, five different models (N-year models) were built concerning accumulation of clinical information: e.g., the 1-year model makes a prediction based only on clinical information from the first year of progression, while the 5-year model makes a prediction based on clinical information from the first to the fifth years of progression. With these year-models, we studied the evolution of prediction performance over the first five years of progression, while investigating which clinical information gains or loses predictive power.

### Database

A database from the Neurology Department of Centro Hospitalar e Universitário de Coimbra (CHUC), whose use for research proposal has been approved by the Ethical Committee of the Faculdade de Medicina da Universidade de Coimbra, was curated by the authors. All methods were performed following the relevant guidelines and regulations. Informed written patient consent was also obtained. The used dataset is comprised of clinical history data from MS patients suffering from RR and SP courses, where the PP patients were excluded, as clinical manifestations of this course are relatively different from the remaining in an initial phase. We chose three different sets of patients, one for SP development prediction and two for disease severeness (one for a prediction in the 6th year of progression another for the 10th), as presented in Table 1.

For the SP course development prediction, the patient selection criteria were the following: (i) the year of the first annotated visit to the clinic was the same or before the diagnosis year (in the case of not having the diagnosis date, we used the estimated onset one), (ii) a minimum of five years of patient tracking, and (iii) the minimum of five annotated visits to the clinic. Additionally, we also selected SP patients whose first annotated visit was after RR diagnosis, but whose SP diagnosis was only after the fifth year of tracking. Despite these patients have not been tracked since onset by the clinic, we guaranteed that the information of all used years still belonged to an RR diagnosed course. Thus, from the selected 187 patients, 21 ($\approx$ 11%) developed into an SP course.

**Table 1:** The used sets of patients for MS disease progression, their selection criteria, patients’ characteristics, number of visits per patients in the first 5 years, and ratio of patients with no year visits for the first 5 years.

Prediction	Number of patients	Less frequent case	Selection criteria	Patient’s characteristics	Visits per patient in first 5 years	Ratio of patients with no year visits in first five years (0–1)
SP development	187	SP developed 21 patients (11%)	i) Tracked since onset diagnosis or with SP diagnosis only after the 5${\mathrm{th}}$ year of tracking;	Gender: 51 men (27%);	1st year: 1.57±0.93;	1st year: 0.00;
			ii) Minimum of 5 years of tracking;	Onset age: 31.10±10.54;	2nd year: 1.25±1.27;	2nd year: 0.36;
			iii) Minimum of 5 annotated visits;	Tracked years: 11.01±8.18;	3rd year: 1.14±1.06;	3rd year: 0.35;
				Annotated years 13.22±4.87;	4${\mathrm{th}}$ year: 1.19±1.03;	4${\mathrm{th}}$ year:0.32;
					5${\mathrm{th}}$ year: 1.28±0.91;	5${\mathrm{th}}$ year: 0.22;
Disease severity in the 6th year	145	Severe cases 38 patients (26%)	i) Tracked since onset diagnosis	Gender: 44 men (28%);	1st year: 1.61±0.96;	1st year: 0.00;
			ii) Minimum of 6 years of tracking;	Onset Age: 30.28±10.89;	2nd year: 1.28±1.20;	2nd year: 0.33;
			iii) Minimum of 5 annotated visits;	Tracked years: 10.67±7.89;	3rd year: 1.14±1.06;	3rd year: 0.32;
				Annotated years 13.82±4.76;	4${\mathrm{th}}$ year: 1.23±1.05;	4${\mathrm{th}}$ year: 0.30;
					5${\mathrm{th}}$ year: 1.37±0.92;	5${\mathrm{th}}$ year: 0.19;
Disease severity in the 10th year	67	Severe cases 30 patients (45%)	i) Tracked since onset diagnosis	Gender: 15 men (27%);	1st year: 1.19±0.47;	1st year: 0.00;
			ii) Minimum of 10 years of tracking;	Onset age: 32.30±11.84;	2nd year: 0.52±0.88;	2nd year: 0.64;
			iii) Minimum of 5 annotated visits;	Tracked years: 15.24±10.35;	3rd year: 0.61±0.74;	3rd year: 0.54;
				Annotated years 15.90±4.83;	4${\mathrm{th}}$ year: 0.75±0.94;	4$^{\mathrm{th}}$ year: 0.51;
					5${\mathrm{th}}$ year: 0.87±0.80;	5$^{\mathrm{th}}$ year: 0.36;

For the disease severity, we selected only patients tracked since diagnosis/onset. We required a minimum of six/ten years of patient tracking, as our disease severity indicators were obtained from the 6th and 10th years. We were able to select 145 and 67 patients, respectively. These two data sets are nested in the SP development one (they are a subset of the 187 patients’ set) and have 57 patients in common (see Supplementary Material for a more detailed explanation). As MS is highly heterogeneous in terms of clinic manifestations, a more severe progression can be related to several aspects, such as an increase in the number of relapses, more severe relapses, or a progressive accumulation of disability. To include all of these possibilities while handling missing annotated visits for some years and possible missing EDSS values, we calculated the mean EDSS from all visits to the clinic (routine and non-routine) that happened in the 6th and 10th years. When those years did not contain any annotated visits for a given patient, we considered one of two possible consecutive years. If the obtained EDSS value was higher than 3, that patient was considered to have a severe disease. Thus, in the 6th year of progression, from the selected 145 patients, 38 (26%) were considered to have a severe disease. In the 10th, from the selected 67 patients, 30 ($\approx$ 45%) were considered to have a severe disease.

Despite each patient usually attends the clinic for routine visits every three or six months, significant gaps are present (missing data), e.g.: no visits were annotated in some years, for some patients. Concerning these gap years, they were intermittent and varied for each patient. Nevertheless, our methodology deals with missing data, as explained in the machine learning pipeline section. The database contains static and dynamic information. Static one is comprised of general information as age, onset age, initial MS manifestations, clinical and exam findings that lead to the diagnosis, such as MRI, CSF analysis, and evoked potentials. Other information, such as concomitant diseases or family history was not used due as these were not available for a significant number of patients. With concern to dynamic information, it is comprised of visits made to the clinic, relapses, treatment and MRI exams. In all annotated visits, the following information was theoretically annotated: if it was a routine or non-routine visit, the EDSS value, each FS score, and symptoms. For all relapses, a CNS evaluation, treatment, if the patient lost its ambulatory capacity, if the patient recovered, the relapse severity, and need for hospitalization were annotated. Missing values are also present for some fields, they are intermittent and varied for each visit, as well as for relapses. All categorical variables were transformed by a one-hot-encoding process.

Medication, including corticosteroid administration during relapses, was not considered as it concerns the physician’s decision and thus, constitutes biased information. Exams, such as data from lumbar puncture for CSF analysis and evoked potentials tests that lead do diagnosis, along with MRI were also excluded due to a large quantity of missing data and/or were not available for a significant number of patients. One can find a complete list of the database available information, along with its usage (used/not used), level of missing values, and static/dynamic role, in Tables S1, S2, and S3 from Supplementary Material. Summarily, we used static information related to age, gender, initial MS manifestations, findings leading to diagnosis, and dynamic information concerning visits (EDSS, FS scores, symptoms) and relapses (CNS evaluation, ambulatory capacity, patient recovery, relapse severity, and hospitalization required).

### Machine learning pipeline

**Figure 2:**
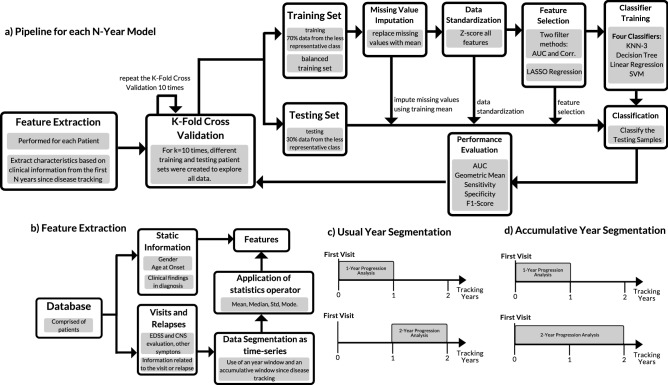
Machine Learning used pipeline: after feature extraction, a k-fold cross validation (with *k*=10) is performed 10 times, which includes missing data imputation, data standardization, feature selection, model training, classification and performance evaluation. The cross validation is performed 10 times. The final performance is the averaged from all runs.

For each N-Year model, the same machine learning pipeline was applied (see Fig. 2a). In short, feature extraction is based on the clinical information from the first *N* years since the patient’s first visit to the clinic, to obtain measurable properties from the disease progression. Then, a group of patients (training set) is selected for missing value imputation, standardization, feature selection, and classifier training. The performance is then evaluated with the remaining patients (testing set), which comprise unseen data by the classifier. This process was performed 100 times, wherein each time, different patients were selected for the training and testing sets. In these 100 executions, we used 10 different k-fold cross-validations (each one with $k=$10) for splitting the training/testing sets, which allowed us to better explore all data and therefore, to make predictions for all patients. The final performance was obtained by averaging the results from all executions.

The first phase consisted of feature extraction, as illustrated in Fig. 2b). Static and dynamic information was used, where the dynamic one suffered a temporal segmentation and was treated as time-series through sliding window analysis. As MS progression is commonly observed in terms of years, data was segmented annually. To each time-window and each source of clinical information, the following statistics were applied: mean, median, standard deviation and mode. It is important to note that each *N*-year model has a different number of features, as more information is available with more tracked years. One can see, in Tables S4, S5 and S6 from Supplementary Material, a complete list of extracted features for each *N*-year model.

It is important to note that besides the usual year segmentation (see Fig. 2c), we also used an accumulative window (see Fig. 2d) by analyzing information starting from the patient’s first visit. The difference between extracted features from those two segmentation windows is solely the used time-window (e.g. an extracted feature with a 2-year usual window uses data from the second year whereas the accumulative one uses the data from the first and second years). The accumulative window allowed to handle part of existing missing data. Despite the database containing information about patients with several years of tracking, there were of gaps of information in some years, as seen in Table 1. There is no guarantee that the patient attended a different clinic or that every field was carefully filled in for each visit, for example. By using an accumulative time-window, the quantity of missing data with feature extraction was significantly reduced, as past information is accounted (e.g., if a given patient does not have an annotated visit in the 3rd year of tracking, an accumulative window can still extract a feature for that year, using the available information from the 1st and 2nd years). Nevertheless, in the case of fields with no annotated value for any past visit for a given patient, it was not possible to extract features for that patient and consequently, missing data was still present.

**Figure 3:**
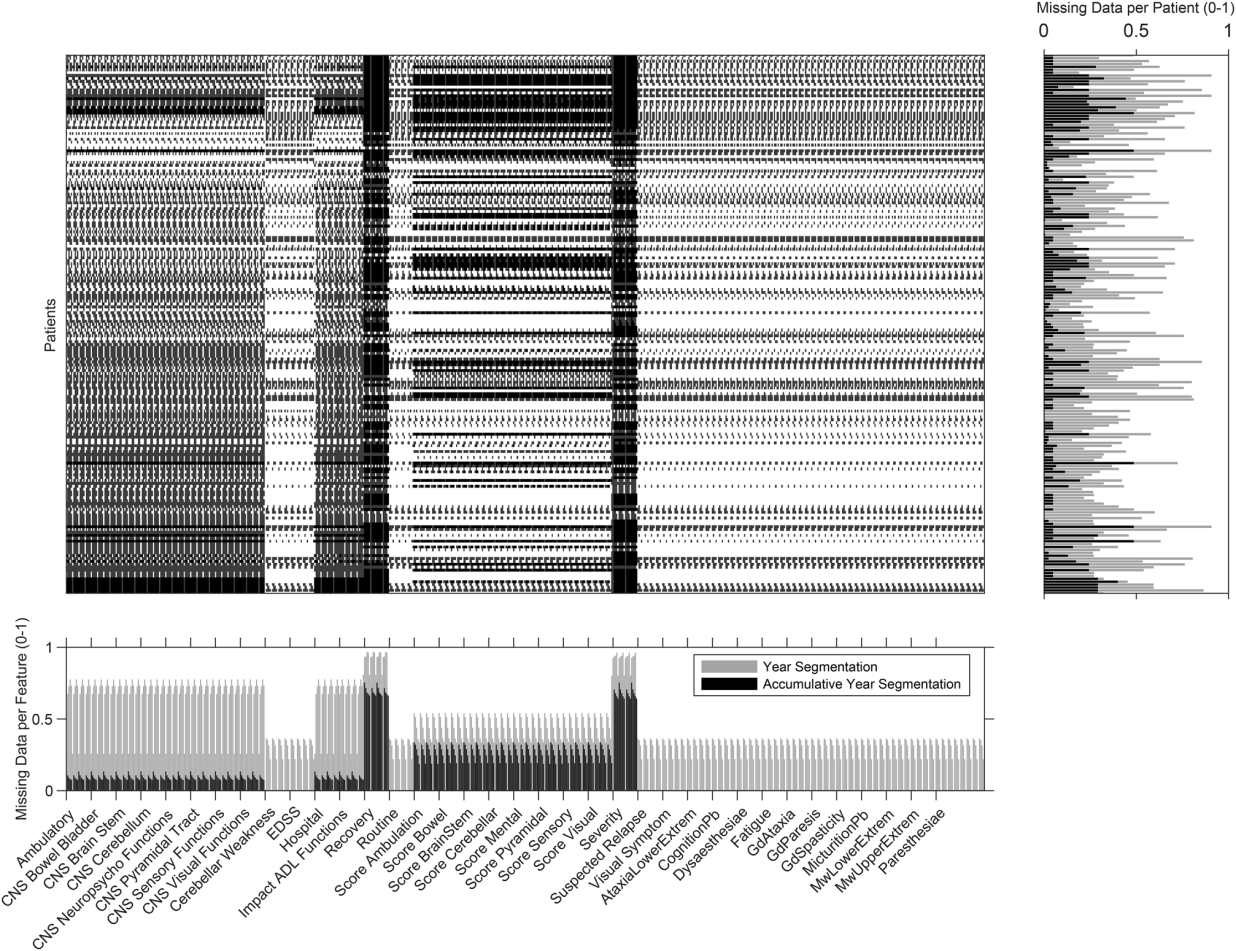
Missing data heatmap concerning features extracted from dynamic information for the first five tracked years, from the patients used in SP development prediction. Missing information per patient and per feature are presented on the right and below, respectively. Accumulative missing data is presented in black while normal segmentation data is presented in gray. In the matrix, patients are organized in rows and features in columns.

Figure 3 shows a missing data heatmap of all extracted features from dynamic information, using the usual segmentation and the accumulative one (see Table S7 from Supplementary Material more detail). It concerns the extracted features from the first five years of progression for each patient regarding relapses and visits to the clinic (in this case, for the patients used in the SP development prediction as it is the largest data set). It is also possible to visualize the ratio of missing data per patient (on the right) and the ratio of missing data per feature (below). With this figure, it is also possible to verify the reduction of missing data with an accumulative analysis. Only a reduced number of patients presented zero missing values. The present spike shape of the graph is explained by the order of the variables: inside each source of clinical information (e.g., CNS Cerebellum evaluation), data is ordered by progression years.

With the obtained features, a modification of the k-fold cross-validation with $k=10$ was performed, which had the goal of exploring all data, as mentioned. Firstly, patients were shuffled randomly and separated into *k* groups, where each group had the real proportion of patients from both classes (SP development/not development or severe/not severe). In each $k\mathrm{th}$ iteration, 70% of patients from the less representative class are selected for the training set, along with an equal number of patients from the other class. The remaining patients comprise the testing data. With this, we not only guaranteed a reasonable number of patients from the less representative class for training but also a balanced set, as it contained an equal number of patients from both classes. It is important to mention that the selected groups of patients for training and testing were different for all *k* iterations since we used the cross-validation strategy for ordering the patients. By repeating this process 10 times, we created 100 different training and testing sets, which allowed us to have a higher degree of certainty on the obtained performance.

The first step, with the training set, is to impute missing values. As the dynamic information-based features were numerical, all values from the training set were imputed with the respective mean. This missing value imputation is performed using the values of all patients. At this stage, we handled this missing data problem as a classic machine learning and not as a time-series one, since each patient constitutes a sample. In our work, missing-data in raw data time-series is handled at the feature extraction stage by using accumulative windows. Then, the data was z-score standardized. The existing missing data was assumed to be from the Missing Completely at Random (MCAR) type, as patients visit, at least, the clinic periodically due to routine visits. Therefore, gaps concerning a complete patient clinic absence are only due to non-annotations. Additionally, all the information fields from visits and relapses did not depend on a determined medical situation or, in other words, these are always extracted. As a consequence, any missing value was assumed to be not related to any of the observed data. One can see the percentage of imputed values, for each feature and each data set, in Tables S7, S8, and S9 from Supplementary Material.

Feature selection was based in two stages: using two filter methods^22^ and an embedded one. Concerning the filter methods: firstly, we selected the best 100 features with Pearson’s linear correlation coefficient and secondly, the best 50 with Area Under the Curve (AUC). Then, the embedded method Least Absolute Shrinkage and Selection Operator (LASSO)^23^ was used to select an optimal set of features. The filter method had the objective of substantially decreasing the number of features and thus, computational power, as it would take the LASSO regression a long period to converge, and therefore, to compute. One can see, in Tables S10, S11, and S12, the complete list of selected features, along with its level of correlation and AUC values.

LASSO does both feature selection and regularization, and thus, enhances prediction accuracy while removing redundancy as it is well-suited for models showing high levels of multicollinearity. Thus, it did not choose the best features individually but rather the optimal set of features acting as a whole. It performs L1 regularization by adding an equal penalty to the absolute value of the magnitude of the coefficient, which results in sparse models, as some become approximately zero due to large penalizations. Consequently, these are eliminated from the model. The cost function of this regression, for *M* points and *P* coefficients, is described in Eq. (1), where $\lambda$ controls the L1 penalty influence, $\mathbf{w }$ is the regression coefficients vector, $\mathbf{y }$ the data to be fitted, $\hat{\mathbf{y }}$ the fitted regression values according to predictors $\mathbf{x }$:

1 $$\begin{aligned} \sum _{i=1}^{M}(y_{i}-\hat{y_{i}})^{2}=\sum _{i=1}^{M}(y_{i}-\sum _{j=1}^{P}w_{j}\times x_{ij})^{2}+\lambda \sum _{j=1}^{P}\left| w_{j} \right| . \end{aligned}$$

If $\lambda =0$, no parameters are eliminated as the regression is equal to the linear regression one. With the increase of $\lambda$, there is a higher regularization action, as more coefficients are set to zero and eliminated.

In this work, we calculated a LASSO regression for a geometric sequence of 100 $\lambda$ values, with only the largest one able to produce a model with 0 features. Then, we selected the $\lambda$ value according to the desired number of features, which were given by a rule of thumb^24^(there should be at least 5 training samples for each feature). Thus, as the training set for SP prediction was comprised of 30 samples (2 classes*0.7*21 SP patients), 5 was the maximum allowed number of features. For disease severeness in the 6th and 10th years, respectively, 10 and 8 were the maximum number of features as the training set was comprised of 53/42 samples (2 classes*0.7*38/30 severe cases). We opted for a rule of thumb instead of performing a more standard cross-validation procedure to get the value of lambda, as the latter did not always converge. In other words, the selected lambda corresponded to a model with 0 features. This might be explained by the small size of our training sets.

Lastly, with the selected features from the training set, four classifiers^25,26^ were trained: a Support Vector Machine (SVM) with a linear kernel, a *k*-Nearest Neighbours (k-NN), a Decision Tree and a Linear Regression.

The *k*-NN classifier^27^ is recognized as lazy because the training process corresponds to data storage. A test sample label is determined by the most prevalent class among the corresponding *k* nearest neighbours. Commonly, the optimal *k* value is obtained experimentally. In this work, $k=3$ was chosen without tuning, as $k=1$ would be too sensitive to noise and, therefore, overfitting. The used distance between neighbours was the Euclidean one.

SVMs^26,28^, in the simplest form, constructs linear decision boundaries for the classification of two-class data. The classification model can be expressed as follows:

2 $$\begin{aligned} f({\mathbf {z}})=sgn\left( {\mathbf {w}}^T{\mathbf {z}}+b\right) . \end{aligned}$$

where ${\mathbf {w}}$ is the normal vector to the decision hyperplane, ${\mathbf {z}}$ the a new sample to be classified, and *b* is a bias term. ${\mathbf {w}}$ and *b* are obtained by minimizing:

3 $$\begin{aligned}&\Psi =\frac{1}{2}||{\mathbf {w}}||^2+C\sum _{i=1}^N\xi _i, \text{ subjected } \text{ to } \end{aligned}$$

4 $$\begin{aligned}&y_i\left( {\mathbf {w}}^T{\mathbf {x}}+b\right) \ge 1-\xi _i;i=1,\cdot\cdot\cdot ,M. \end{aligned}$$

where $\{{\mathbf {x}}_i,y_i\}$ (with $i=1,\cdot\cdot\cdot ,M$) is the training data, being ${\mathbf {x}}_i$ input feature vectors and $y_i\in \{-1,+1\}$ the class labels. $\xi$ is a quantification of the degree of misclassification, and *C* defines the influence of $\xi$ on the minimization criterion $\Psi$.

Linear regression^26^ is the simplest linear model for regression, which involves a linear combination of the input variables, where $w_{n}$ is the regression coefficient value concerning the feature $x_{n}$, as shown in equation (5):

5 $$\begin{aligned} f(\mathbf{z })= w_{0}+\sum _{i=1}^{P}w_{i}z_{i}. \end{aligned}$$

Decision Tree classifiers^29^ learn from data a simple representation with a set of if-then-else decision rules. With this set, it is possible to make complex decisions. By separating the dataset into cluster regions according to these if-then-else decisions, the tree is incrementally developed and the final output is a tree with decision nodes and leaf nodes.

After having our classifiers trained, an analogous procedure is performed to the testing set: the selected features are the ones obtained from the training set. To them, their missing values are imputed using the obtained mean values from the training set, and the z-score process is based on the corresponding features’ mean and standard deviation from the training set. Finally, performance is evaluated by comparing the output of the trained classifiers on the testing set with the real labels.

This comparison enables the construction of a confusion matrix, where we obtain four measurements: true positives (TP): number of samples correctly classified as the positive; false positives (FP): number of samples classified as positive that are negative; true negatives (TN): number of samples correctly classified as negative; and false negatives (FN): number of samples classified as negative that are positive. With the confusion matrix, we can calculate the following metrics: geometric mean, sensitivity, specificity, and F1-Score. We also obtain the AUC as it is a common metric in medical problems. It represents the area under the Receiver Operating Characteristics (ROC) curve, which is created by plotting the sensitivity, also known as the True Positive Rate (TPR) against the false positive rate (FPR), concerning a varying decision threshold.

Sensitivity (or recall) and specificity provide the proportion of actual positives correctly identified and the proportionality of negatives correctly identified. By other words and by considering the SP course prediction, sensitivity is the proportion of SP courses correctly predicted while specificity is the proportion of non-SP development correctly predicted. Finally, the geometric mean is presented as it can be considered a more adequate measure than accuracy, as the dataset is significantly imbalanced (for example, patients that developed SP comprise only $\approx$10% of all cases). Thus, sensitivity, specificity, and geometric mean are given by:

6 $$\begin{aligned} Sensitivity= & {} \frac{TP}{TP+FN}, \end{aligned}$$

7 $$\begin{aligned} Specificity= & {} \frac{TN}{TN+FP}, \end{aligned}$$

8 $$\begin{aligned} Geometric Mean= & {} \sqrt{Sensitivity * Specificity}. \end{aligned}$$

Furthermore, $F_{1}$ Score is also shown, as it accounts for sensitivity and precision where the last one corresponds to the proportion of correctly predicted positives among all cases identified as positives. In other words, precision provides the proportion of correctly predicted SP/severe cases among all predicted as such. As this is a medical problem where each course/prognostic might influence the medication intake, it is also important to note its relevance to this particular problem. Precision and $F_{1}$ Score are given by:

9 $$\begin{aligned} Precision= & {} \frac{TP}{TP+FP}, \end{aligned}$$

10 $$\begin{aligned} F_{1}\text {-}Score= & {} \frac{2*Precision*Sensitivity}{Precision+Sensitivity}. \end{aligned}$$

Concerning implementation, we used MATLAB R2018b for all stages of this work. All functions are built-in in MATLAB with the exception of the standardization^30^ and missing data imputation^31^. For calculating correlation coefficient and AUC, we used *corr* and *perfcurve* functions, respectively. For LASSO, we used *lasso* function. For the training the classifier models, we used *fitcknn*, *fitctree*, *fitlm*, and *fitcsvm* functions for the KNN-3, the decision tree, the linear regression and the SVM, respectively. To predict new samples, we used *predict* function. Finally, we used *cvpartition* for the cross validation.

## Results and discussion

**Table 2:** Obtained results for the two predictions: SP development/not development and disease severity. For each prediction, all classifier performances for all N-year models are presented, where the best are in bold.

		SP Development/Not Development				Disease Severity in the 6th Year Progression				Disease Severity in 10th Year Progression			
		KNN-3	Decision Tree	Linear Regression	SVM	KNN-3	Decision Tree	Linear Regression	SVM	KNN-3	Decision Tree	Linear Regression	SVM
1-Year Model	AUC	0.76±0.05	0.65±0.10	0.79±0.06	**0.81±0.06**	0.74±0.06	0.74±0.07	0.80±0.06	**0.80±0.06**	0.67±0.09	0.57±0.09	0.67±0.09	**0.73±0.07**
	Geometric Mean	0.67±0.05	0.61±0.08	0.71±0.06	**0.72±0.06**	0.70±0.06	0.69±0.08	0.71±0.05	**0.71±0.06**	0.61±0.08	0.55±0.09	0.62±0.08	**0.65±0.08**
	Specificity	0.60±0.06	0.55±0.11	0.65±0.07	**0.65±0.08**	0.66±0.09	0.64±0.12	0.73±0.06	**0.69±0.06**	0.61±0.12	0.61±0.13	0.67±0.09	**0.63±0.12**
	Sensitivity	0.75±0.11	0.70±0.17	0.80±0.14	**0.81±0.13**	0.74±0.10	0.76±0.12	0.70±0.10	**0.73±0.11**	0.63±0.14	0.51±0.14	0.58±0.14	**0.67±0.11**
	F1-Score	0.13±0.02	0.11±0.03	0.15±0.03	**0.16±0.03**	0.36±0.06	0.35±0.07	0.39±0.06	**0.37±0.06**	0.54±0.09	0.46±0.10	0.54±0.10	**0.58±0.08**
2-Year Model	AUC	0.81±0.08	0.70±0.09	0.82±0.08	**0.86±0.07**	0.80±0.05	0.83±0.06	0.87±0.04	**0.89±0.03**	0.66±0.09	0.61±0.07	0.71±0.07	**0.71±0.07**
	Geometric Mean	0.73±0.08	0.66±0.08	0.76±0.08	**0.76±0.08**	0.75±0.05	0.77±0.08	0.82±0.06	**0.82±0.06**	0.58±0.10	0.55±0.09	0.66±0.07	**0.63±0.08**
	Specificity	0.74±0.06	0.65±0.09	0.78±0.05	**0.77±0.05**	0.68±0.06	0.72±0.09	0.82±0.04	**0.81±0.05**	0.48±0.13	0.52±0.15	0.63±0.11	**0.54±0.13**
	Sensitivity	0.73±0.14	0.69±0.16	0.74±0.14	**0.76±0.14**	0.83±0.11	0.83±0.14	0.83±0.11	**0.84±0.11**	0.73±0.15	0.62±0.17	0.70±0.12	**0.75±0.12**
	F1-Score	0.18±0.05	0.13±0.04	0.21±0.05	**0.20±0.05**	0.40±0.05	0.44±0.09	0.53±0.06	**0.53±0.07**	0.55±0.09	0.50±0.10	0.59±0.07	**0.59±0.07**
3-Year Model	AUC	0.73±0.09	0.65±0.12	0.78±0.09	**0.83±0.09**	0.81±0.05	0.82±0.07	0.89±0.03	**0.90±0.03**	0.68±0.08	0.65±0.08	0.73±0.07	**0.75±0.07**
	Geometric Mean	0.70±0.10	0.65±0.11	0.73±0.09	**0.73±0.09**	0.76±0.06	0.77±0.09	0.84±0.05	**0.83±0.04**	0.61±0.10	0.58±0.09	0.67±0.07	**0.69±0.08**
	Specificity	0.70±0.06	0.62±0.11	0.75±0.06	**0.72±0.06**	0.73±0.04	0.75±0.09	0.84±0.02	**0.78±0.06**	0.59±0.15	0.59±0.16	0.72±0.07	**0.66±0.11**
	Sensitivity	0.73±0.18	0.69±0.19	0.72±0.18	**0.77±0.18**	0.80±0.12	0.80±0.16	0.85±0.11	**0.88±0.10**	0.67±0.16	0.61±0.17	0.63±0.12	**0.73±0.12**
	F1-Score	0.16±0.04	0.13±0.05	0.19±0.05	**0.17±0.04**	0.42±0.05	0.45±0.09	0.57±0.06	**0.51±0.06**	0.56±0.10	0.52±0.10	0.59±0.09	**0.62±0.08**
4-Year Model	AUC	0.79±0.08	0.65±0.10	0.79±0.07	**0.85±0.06**	0.85±0.05	0.85±0.07	0.92±0.03	**0.92±0.04**	0.72±0.08	0.70±0.10	0.77±0.08	**0.80±0.08**
	Geometric Mean	0.73±0.08	0.65±0.10	0.75±0.08	**0.77±0.09**	0.78±0.07	0.79±0.07	0.86±0.05	**0.84±0.04**	0.66±0.10	0.64±0.10	0.71±0.10	**0.75±0.08**
	Specificity	0.74±0.06	0.66±0.12	0.78±0.06	**0.77±0.07**	0.82±0.04	0.77±0.08	0.89±0.04	**0.82±0.05**	0.70±0.11	0.71±0.15	0.81±0.08	**0.76±0.09**
	Sensitivity	0.74±0.16	0.66±0.18	0.72±0.13	**0.78±0.16**	0.74±0.13	0.82±0.14	0.83±0.09	**0.87±0.08**	0.65±0.16	0.61±0.17	0.64±0.15	**0.74±0.13**
	F1-Score	0.18±0.04	0.14±0.05	0.21±0.05	**0.21±0.05**	0.49±0.08	0.48±0.09	0.63±0.07	**0.56±0.07**	0.59±0.11	0.57±0.11	0.64±0.12	**0.68±0.09**
5-Year Model	AUC	0.84±0.08	0.77±0.13	0.81±0.06	**0.86±0.07**	0.87±0.05	0.86±0.09	0.94±0.03	**0.94±0.04**	0.79±0.08	0.77±0.10	0.81±0.07	**0.85±0.07**
	Geometric Mean	0.79±0.09	0.74±0.12	0.75±0.11	**0.77±0.13**	0.77±0.08	0.84±0.09	0.87±0.06	**0.88±0.05**	0.72±0.10	0.70±0.11	0.74±0.09	**0.78±0.08**
	Specificity	0.77±0.06	0.77±0.13	0.82±0.06	**0.80±0.06**	0.86±0.04	0.87±0.09	0.93±0.03	**0.88±0.04**	0.75±0.11	0.80±0.16	0.85±0.09	**0.79±0.09**
	Sensitivity	0.82±0.17	0.74±0.20	0.71±0.20	**0.75±0.20**	0.71±0.14	0.83±0.16	0.81±0.12	**0.87±0.10**	0.72±0.14	0.63±0.15	0.66±0.13	**0.77±0.13**
	F1-Score	0.22±0.05	0.22±0.08	0.24±0.07	**0.23±0.06**	0.51±0.08	0.61±0.11	0.71±0.08	**0.65±0.07**	0.66±0.12	0.64±0.12	0.68±0.11	**0.72±0.09**

**Figure 4:**
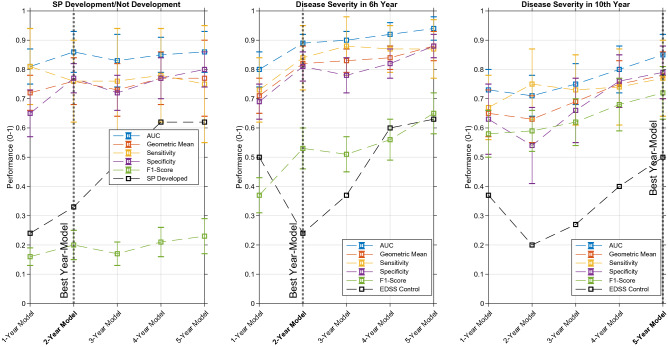
The SVM classifier performance in each N-year model for each prediction. The best performances for SP development was achieved in the 2-year model. Concerning disease severity, the best performances were achieved for the 2-year model and the 5-year model, concerning the 6th and 10th years, respectively.

In Table 2, one can see the obtained results for the three predictions, one for SP development, and two for disease severity, for each N-year model. In the overall scenario, we believe the SVM was the classifier that achieved the best results. Linear regression and linear SVM (linear) outperformed the KNN-3 and Decision Tree (non-linear) classifiers, which we considered normal since we used LASSO for feature selection. With Fig. 4, it is possible to see the SVM performance evolution over time for the three predictions. Besides, in both cases, a sort of control performance is also plotted: SP developed and EDSS control (black lines). For the first, it concerns the accumulative ratio of patients that developed SP in the Nth progression year within all patients that developed SP. For the disease severity, it concerns the ratio of patients that had a mean EDSS>3 in the Nth progression year within all patients with a severe disease (mean EDSS>3 in the 6/10th year of progression). These can be compared with the obtained models sensitivity.

In SP development prediction, an increase in the Geometric Mean and $F_{1}$-Score metrics is visible over time. Nevertheless, the AUC metric has a peak performance in the 2-year model. Due to this and despite the best overall performance may be obtained in the 5-year model, it is desirable to achieve a good prediction with the lowest number of progression years. Thus, we considered the 2-year model as the best for predicting SP development, with an AUC of $0.86\pm 0.07$, a geometric mean of $0.76\pm 0.08$, a sensitivity of $0.76\pm 0.14$, a specificity of $0.77\pm 0.05$, a precision of $0.12\pm 0.03$, and an $F_{1}$-Score of $0.20\pm 0.05$. Concerning control performance, sensitivity outperformed it for all years. However, this performance difference is more significant in the first three years.

Regarding disease severity prediction for the 6hth year, it is clear that performance increases monotonically over time for almost all metrics. As it is also desirable to have a good performance in the minimum years of progression, we believe the 2-year model is the most adequate, with an AUC of $0.89\pm 0.03$, a geometric mean of $0.82\pm 0.06$, a sensitivity of $0.84\pm 0.11$, a specificity of $0.81\pm 0.05$, a precision of $0.39\pm 0.06$, and an $F_{1}$-Score of $0.53\pm 0.07$.

For disease severity regarding the 10th year, the performances are not as good as for the 6th, which can be considered natural as it concerns a longer prediction period. Similarly, the prediction performance increases with time. As in the early years it may be more important to predict an earlier future, we believe that, for predicting the severeness in the 10th year, we can choose a later year-model. Thus, we selected the 5-year model as the best, which concerns a prediction on the following 5 years and a satisfactory performance is achieved: an AUC of $0.85\pm 0.07$, a geometric mean of $0.78\pm 0.08$, a sensitivity of $0.77\pm 0.13$, a specificity of $0.79\pm 0.09$, a precision of $0.68\pm 0.10$, and an $F_{1}$-Score of $0.72\pm 0.09$. Concerning control performance, sensitivity outperformed significantly it for all years, for both disease severity predictions.

When comparing both predictions, we believe disease severity was more successful than SP development, which might be explained by the unpredictability of the SP course onset. As seen with the control group, more than 60% of SP patients developed this course until the 5th year of progression. Additionally, it is also the problem with the highest class imbalance (11%) and with the lowest number of samples in the less representative class.

When comparing our results with Zhao et al.^13^, that considered a severe case when the EDSS increases 1.5 up to 5 years after baseline visit, our 2-year model outperformed simultaneously in terms of sensitivity, specificity, and accuracy (in our case, geometric mean). Concerning Law et al.^19^, our results are better but it is very important to note that their window of analysis is significantly different, as they tracked patients for up to a maximum of 24 months and made predictions, throughout time, based on a six-month window of advance. Seccia et al.^17^, using a 2-year window and a linear SVM classifier, presented better results than our 2-year model, concerning accuracy, specificity, and sensitivity, but lower in terms of precision. Additionally, with a Long Short-Term Memory (LSTM) classifier, they were able to significantly outperform our work in terms of precision, accuracy, and specificity, but underperformed regarding sensitivity. Summarily, it is important to note that these comparisons are limited to the way authors not only obtain a benign/malign distinction but also to the used data set and their window of analysis. Therefore, we would like to highlight that our number of patients is reduced, regarding the previously compared works.

Regarding $F_{1}$-Score, despite the lower results for the SP development and disease severity in the 6th progression year, we believe it is an important measure. It demonstrates the limitation of the presented methods^17^. As precision represents the patients correctly classified with SP/severe among all classified as such, and since this misclassification might bring consequences to the patient in terms of wrong medication intake, it is necessary to stress this difficulty^17,19^. Nevertheless, the data imbalance scenario influences this measure significantly, as it decreases with higher class imbalance. Besides achieving high performances, as the case of the LSTM trained by Seccia et al.^17^, it is important to understand which predictors contribute to it, to provide knowledge to a physician. Knowing the predictors’ importance might be hard to achieve when using an LSTM, as these models are more complex and their learning phase requires a large quantity of data. In fact, Pellegrini et al.^18^ confirmed their relatively poor performance in modelling MS progression with the inconsistencies that were found within their predictors’ importance, for all models.

**Figure 5:**
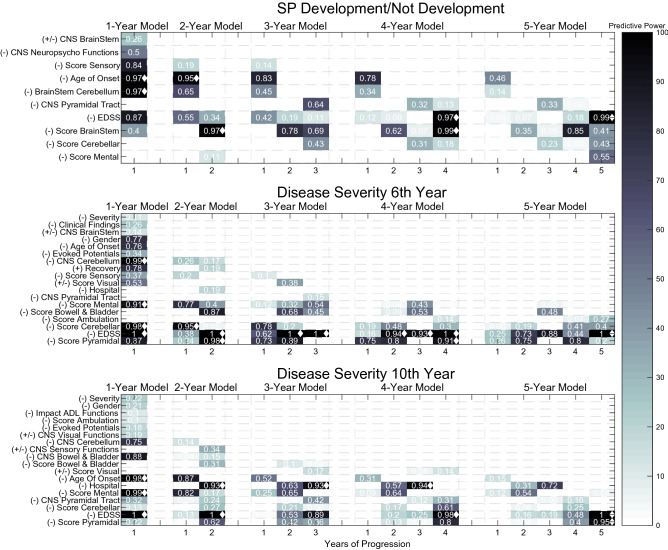
The selected features for SP development/not development and disease severity, for all year models. Color represents predictive power, calculated by the presence of each clinical source among all $k*10=100$ iterations. The signs are related to the clinical feature prognostic influence: (+) represents a good prognostic and (−) represents a bad prognostic. The diamond marker represents predictive power above 0.90.

Figure 5 shows the selected features for both predictions, for each of the 5-year models. Colour represents predictive power, which was calculated by summing the presence of each clinical source among all $10*k=100$ iterations (0 for no appearance in 100 runs; 1 for 100 appearances in the 100 runs). Clinical sources with less than 10 presences were discarded, as they were considered non-significant (predictive power below 0.10). Furthermore, due to its heterogeneity in terms of mathematical operations, segmentation (accumulative or normal), and due to clinical interpretability issues, we only show its clinical source and correspondent year of progression. Additionally, the clinical sources were also chronologically ordered concerning their appearance, to have a vision towards evolution over time. Furthermore, by inspecting the features correlation value (if positive or negative) with the label, it was possible to determine their influence towards a favourable (+) or an unfavourable progression outcome (−). For example: with a positive correlation, a feature was considered to influence SP development or a severe case (−); with a negative correlation, a feature was considered to influence towards not developing SP course or towards a benign form (+). The diamond marker represents features with predictive power above 0.90.

Regarding SP development, the selected features were mostly related to the EDSS, FS scores (sensory, brainstem, cerebellar, and mental) annotated in visits, and affected functions of the CNS (pyramidal tract, neuropsychological, and brainstem) annotated during relapses. Age at onset was also significantly present. While the majority was present throughout all year models, the cerebellar score and CNS pyramidal tract only started to appear later, since the 3-year model. On the other hand, damage on the neuropsychologic functions and brainstem during relapses were only considered predictive in the 1-year model, and the sensory functional system was considered predictive until the 3-year model. The mental functional score was only significantly predictive in the 5-year model. Age at onset, the EDSS, BrainStem FS score and initial manifestations on the BrainStem and/or cerebellum were the features that achieved a predictive power above 0.90, in at least one year-model.

When analysing its influence, the majority was associated with a bad prognosis. However, affected brainstem functions during relapses may not be a sign of a bad outcome. This relation does not seem to be straightforward: damage to brainstem functions was only associated with a bad prognosis concerning its standard deviation values (no other CNS brainstem feature was selected with a different mathematical operator). In other words, when relapses affected brainstem functions intermittently. This may suggest that relapses affecting brainstem functions may be characteristic of different courses, depending on their frequency. Summarily, these findings led us to conclude that, patients whose relapses affect brainstem functions frequently/rarely are more likely to maintain an RR course, while the ones whose relapses affect these functions intermittently are likely to develop an SP course.

With concern about disease severity and the selected features, the case seems to be more complex. For both predictions (6th and 10th, respectively) in the first years of progression, the number of clinical sources is high and also tends to decrease over time, from about 14/14 in the 1-year model to 6/7 in the 5-year model. Again, the selected features were mostly related to the EDSS, FS scores and affected functions of the CNS during relapses. FS like the mental, cerebellar, pyramidal, the bowel and bladder, and the visual one have predictive power in both predictions. The sensory score concerned only the 6th progression year. Regarding the affected functions of the CNS during relapses, brainstem functions were only found to be predictive for the 6th tracking year, while bowel and bladder, sensory and visual functions were only found to be predictive for the 10th tracking year. In both, affected functions during relapses concerning cerebellum and pyramidal tract were considered important. Some static features appeared as predictive, as the case of age of disease onset, gender, initial findings of MS in evoked potentials exam, and gender. However, these presented reduced importance, except for age at onset. Other features were also present, related to relapses severity/recovery, as the case of a patient hospitalization due to a relapse. In terms of a significantly high predictive power, CNS Cerebellum, Cerebellar, mental and pyramidal FS scores, and EDSS achieved values above 0.90, for at least 1 year model, for predicting disease severity at the 6th tracking year. Concerning the 10th tracking year, age of onset, relapse hospitalization, pyramidal and mental scores, and the EDSS were the ones achieving a predictive value above 0.90, for at least one year model.

It is interesting to see that, when one investigates which features are characteristic from the first and last years of progression, there is a coherence in both predictions. The pyramidal, cerebellar and mental FS, and EDSS are the dominant features in the last year models. In short, we believe the most significant difference between the 6th and the 10th prediction, is their feature difference in the early year-models. Female gender was associated with a favourable course, which supports Hawkins et al.^10^ findings. An earlier age of onset was also associated with a favourable course^9,10^.

Most of these features were also defined as relevant in other studies: pyramidal involvement, cerebellar, optic neuritis, sensory disturbances, motor symptoms, and sphincter involvement are examples of it^9,10^. Also, clinical information as the Mental FS Score was included in our models. It is relevant to highlight that different studies suggest different features and even though some present coincident results, others suggest opposite conclusions.

Regarding their influence into severe/not severe cases, both predictions presented coherent results. Any increase in the EDSS or in any FS score was considered severe, as well as the majority of CNS functions affected during relapses. Relapses’ affected CNS visual and sensory functions may be associated with a good patient outcome. Again, damage on CNS visual, sensory and brainstem functions during relapses is not a straightforward relation: patients who suffer from related symptoms in relapses frequently or rarely are more likely to have a non-severe progression, while the ones who suffer from them intermittently are likely to have a more severe progression.

## Conclusion

This work can be considered as a proof-of-concept concerning the possibility of predicting MS progression by using a machine-learning approach. This procedure allowed to simulate a real hospital situation while handling missing values, where the existing data is used to training a model and it is ready to test new upcoming patients whose progression development is unknown. Additionally, it also provided a disease progression analysis through the accumulation of information based on consecutive years. This procedure provided a different understanding, as it concerned the evolution of the predictors’ importance and their corresponding performance.

Concerning relevant clinical information, EDSS, functional systems and affected CNS functions during relapses are among the relevant ones, which coincide with similar studies. Additionally, in the first progression years, the number of features was higher and tended to decrease over time, which supports Bengt Skoog et al.^16^ findings. Despite Reynders et al.^12^ conclusions, features as age at onset and gender were considered significant predictors in our work. Nevertheless, we believe this might not exclude the possibility of their interaction with the disease phenotype.

Despite the low obtained F1-Score values for the SP development prediction, as precision was relatively reduced, the authors believe a machine learning approach can be a powerful study, as it can, in the future, simulate a real-life situation by offering the physician a second opinion^17^. Furthermore, since our data sets were reduced in number of patients and contained a significant amount of missing data, we believe this study results must be confirmed in larger databases that do not have large missing data problems. Future work concerns the usage of more information, as MRI examination, and the use and consequent comparison with different disease severeness criteria.

## Supplementary information

Supplementary Information.
