# Supplementary material for: Prediction of disease progression and outcomes in multiple sclerosis with machine learning
Source: Sci Rep. 2020 Dec 3;10:21038. doi: 10.1038/s41598-020-78212-6 (PMC7713436; doi:10.1038/s41598-020-78212-6)
Supplement: Supplementary file 1 — Supplementary Information. [file 41598_2020_78212_MOESM1_ESM.pdf]

# Prediction of Disease Progression and Outcomes in Multiple Sclerosis with Machine Learning

**Mauro F. Pinto[1,*], Hugo Oliveira[1], Sónia Batista[2], Luís Cruz[3], Mafalda Pinto[3], Inês Correia[2], Pedro Martins[1], and César Teixeira[1]**

[1]University of Coimbra, Centre for Informatics and Systems of the University of Coimbra, Department of Informatics Engineering
[2]Neurology Department of Centro Hospitalar e Universitário de Coimbra
[3]Functional Unit of Neuroradiology, Medical Imaging Department, Centro Hospitalar e Universitário de Coimbra
[*]mauropinto@dei.uc.pt

## Supplementary Material

### Nested Data Sets Explanation

Not 100% patients from the 67 set are included in the 145 one. The latter is due to the occurrence of missing data (missing visits and/or EDSS values) in the years used for calculating the disease severity indicator, in both sets. By not accounting this missing data factor, the data sets would have been completely nested and would comprise 158 and 74 patients for the disease severity at the 6$^{th}$ and 10$^{th}$ tracking years, respectively. Consequently, we removed 13 patients from the 158 set and 7 patients from the 74 set. These two sets have 57 patients in common, which comprises 85% of the smaller set.

### Database Available Information

In Tables S1, S2, and S3, we present all information available in the database. All variables are ordered according to the original database order. We also show, for the three data sets, its level of missing data and for how many patients it is available. With these, one can understand the use of information from *Identification*, *Visits*, and *Relapses* tabs, as these are the ones that contain information for more than 95% of patients, in all data sets.

Information concerning *CSF* examination and *MRI* is also available for a significant number of patients. Nevertheless, these present a significant number of features with a high quantity of missing values. Furthermore, the number of MRI's per patient is 3.67±2.45, 3.94±2.48, and 3.73±2.67 for the SP development, and disease severity at 6$^{th}$ and 10$^{th}$ tracking years, respectively. This number may be reduced when compared to the available number of relapses per patient: 5.21±4.77, 5.34±4.98, and 7.06±6.07.

| Variable | Used | Missing Data Ratio (0-1) | | | Description |
|---|---|---|---|---|---|
| | | **SP Development Prediction** | **Disease Severity in the 6th Year** | **Disease Severity in the 10th Year** | |
| **Identification (Static)** | | **available for 100% patients** | **available for 100% patients** | **available for 100% patients** | |
| Patient ID | Yes | 0.00 | 0.00 | 0.00 | identification of each patient; |
| Gender | Yes | 0.00 | 0.00 | 0.00 | - |
| Age of Onset | Yes | 0.00 | 0.00 | 0.00 | calculated using Birth Date and Diagnosis Date; |
| Deceased | No | 0.00 | 0.00 | 0.00 | - |
| Supratentorial | Yes | 0.00 | 0.00 | 0.00 | binary (yes/no): initial manifestations of MS related to the supratentorial region; |
| Optic Pathways | Yes | 0.00 | 0.00 | 0.00 | binary (yes/no) : initial manifestations of MS related to optic pathways; |
| Brainstem-Cerebellum | Yes | 0.00 | 0.00 | 0.00 | binary (yes/no): initial manifestations of MS related to the brainstem and/or cerebellum; |
| Spinal Cord | Yes | 0.00 | 0.00 | 0.00 | binary (yes/no): initial manifestations of MS related to the spinal cord; |
| Progression from Onset | No | 0.00 | 0.00 | 0.00 | binary (yes/no): if progressive deterioration since onset was observed; |
| Clinical findings | Yes | 0.00 | 0.00 | 0.00 | binary (yes/no): clinical evidence in the MS initial manifestations; |
| MRI | Yes | 0.00 | 0.00 | 0.00 | binary (yes/no): initial MS manifestations visualized in MRI scans (lesions); |
| Evoked Potentials | Yes | 0.00 | 0.00 | 0.00 | binary (yes/no): initial MS manifestations visualized in the evoked potentials test; |
| CSF | Yes | 0.00 | 0.00 | 0.00 | binary (yes/no): initial MS manifestations visualized in the lumbar puncture exam; |
| Family History | No | 0.00 | 0.00 | 0.00 | categorical field (-1, 1, 0, 1, 2): family history of diseases (not MS specific); |
| Birth Date | Yes | 0.00 | 0.00 | 0.00 | used to calculate Age of MS onset; |
| RR Diagnosis Date | Yes | 0.03 | 0.03 | 0.03 | used to calculate Age of MS onset; |
| Date of Onset | No | 0.00 | 0.00 | 0.00 | - |
| SP Diagnosis Date | No | 0.00 | 0.00 | 0.00 | - |
| | | | | | |
| **Family History (Static)** | | **available for 6% patients** | **available for 0% patients** | **available for 5% patients** | |
| Patient ID | No | 0.00 | 0.00 | 0.00 | - |
| Relationship | No | 0.00 | 0.00 | 0.00 | - |
| is MS | No | 0.00 | 0.00 | 0.00 | binary (yes/no): if the disease is MS; |
| Disease Name | No | 0.00 | 0.00 | 0.00 | the disease name, if not MS; |
| | | | | | |
| **Evoked Potentials (Static)** | | **available for 25% of patients** | **available for 30% of patients** | **available for 34% of patients** | |
| Patient ID | No | 0.00 | 0.00 | 0.00 | - |
| Exam Date | No | 0.00 | 0.00 | 0.00 | - |
| Latency Right Result | No | 0.00 | 0.00 | 0.00 | binary (yes/no): manifestations of latency in the right side; |
| Latency Left Result | No | 0.06 | 0.07 | 0.04 | binary (yes/no): manifestations of latency in the left side; |
| | | | | | |
| **CSF (Static)** | | **available for 67% of patients** | **available for 72% of patients** | **available for 78% of patients** | |
| Patient ID | No | 0.00 | 0.00 | 0.00 | - |
| Exam Date | No | 0.00 | 0.00 | 0.00 | - |
| Result normal/abnormal | No | 0.45 | 0.41 | 0.29 | binary (normal/abnormal): the CSF analysis results; |
| Result typical/atypical | No | 0.52 | 0.50 | 0.37 | binary (typical/atypical): when CSF analysis results are abnormal, if they are MS typical/atypical; |
| Oligoclonal Banding | No | 0.02 | 0.03 | 0.02 | binary (yes/no): detection of oligoclonal bands; |
| Matched OCB in Serum | No | 0.23 | 0.27 | 0.18 | binary (yes/no): when detected oligloconal bands, if these were paired in serum; |

**Table S 1.** Database information concerning identification, family history, and CSF and Evoked Potentials examinations. These exams' information was considered static, as it is related to the patient MS diagnosis.

| Variable | Used | Missing Data Ratio (0-1) | | | Description |
|---|---|---|---|---|---|
| | | SP Development Prediction | Disease Severity in the 6th Year | Disease Severity in the 10th Year | |
| **Visits (Dynamic)** | | **available for 100% patients** | **available for 100% patients** | **available for 100% patients** | |
| Patient ID | Yes | 0.00 | 0.00 | 0.00 | - |
| Visit Date | Yes | 0.00 | 0.00 | 0.00 | - |
| Administrative | No | 0.00 | 0.00 | 0.00 | binary (yes/no): non-presential visit to annotate midterm clinical information between routine visits; |
| Routine | Yes | 0.00 | 0.00 | 0.00 | binary (yes/no): routine visit; |
| Suspected Relapse | No | 0.00 | 0.00 | 0.00 | binary (yes/no): not an expected visit, as the patient suspected a relapse; |
| Phone | No | 0.00 | 0.00 | 0.00 | binary (yes/no): non-presential visit, with phone call; |
| Retrospective | No | 0.00 | 0.00 | 0.00 | binary (yes/no): a visit registered retrospectively; |
| Score Pyramidal | Yes | 0.19 | 0.20 | 0.24 | numeric (0-6): Pyramidal FS score; |
| Score Cerebellar | Yes | 0.19 | 0.20 | 0.24 | numeric (0-5): Cerebellar FS score; |
| Cerebellar Weakness | Yes | 0.00 | 0.00 | 0.00 | binary (yes/no): MS manifestations of cerebellar weakness; |
| Score BrainStem | Yes | 0.19 | 0.20 | 0.24 | numeric (0-5): Brain Stem FS score; |
| Score Sensory | Yes | 0.19 | 0.20 | 0.24 | numeric (0-6): Sensory FS score; |
| Score Bowel & Bladder | Yes | 0.19 | 0.20 | 0.24 | numeric (0-6): Bowel & Bladder FS score; |
| Score Visual | Yes | 0.19 | 0.20 | 0.24 | numeric (0-6): Visual FS score; |
| Visual Symptoms | Yes | 0.00 | 0.00 | 0.00 | binary (yes/no): visual symptoms; |
| Score Mental | Yes | 0.19 | 0.20 | 0.24 | numeric (0-5): Mental FS score; |
| Score Ambulation | Yes | 0.19 | 0.20 | 0.24 | numeric (0-12): Ambulation FS score; |
| gdAtaxia | Yes | 0.00 | 0.00 | 0.00 | binary (yes/no): manifestations of gait disturbances related to ataxia; |
| dysaesthesiae | Yes | 0.00 | 0.00 | 0.00 | binary (yes/no): manifestations of dysaesthesiae; |
| ataxiaLowerExtrem | Yes | 0.00 | 0.00 | 0.00 | binary (yes/no): manifestations of ataxia in the lower extremities; |
| paresthesiae | Yes | 0.00 | 0.00 | 0.00 | binary (yes/no): manifestations of paresthesiae; |
| cognitionPb | Yes | 0.00 | 0.00 | 0.00 | binary (yes/no): manifestations of perturbances in cognition; |
| gdParesis | Yes | 0.00 | 0.00 | 0.00 | binary (yes/no):manifestations of gait disturbances related to paresis; |
| gdSpasticity | Yes | 0.00 | 0.00 | 0.00 | binary (yes/no): manifestations of gait disturbances related to spasticity; |
| mwUpperExtrem | Yes | 0.00 | 0.00 | 0.00 | binary (yes/no): manifestations of muscular weakness in the upper extremities; |
| micturitionPb | Yes | 0.00 | 0.00 | 0.00 | binary (yes/no): manifestations of perturbances in micturition; |
| fatigue | Yes | 0.00 | 0.00 | 0.00 | binary (yes/no): manifestations of fatigue; |
| mwLowerExtrem | Yes | 0.00 | 0.00 | 0.00 | binary (yes/no): manifestations of muscular weakness in the lower extremities; |
| moodPb | Yes | 0.00 | 0.00 | 0.00 | binary (yes/no): manifestations of mood perturbances; |
| EDSS | Yes | 0.00 | 0.00 | 0.00 | numerical (0-10): the EDSS value; |
| | | | | | |
| **Relapses (Dynamic)** | | **available for 97% of patients** | **available for 96% of patients** | **available for 100% of patients** | |
| Patient ID | Yes | 0.00 | 0.00 | 0.00 | - |
| Relapse Date | Yes | 0.00 | 0.00 | 0.00 | - |
| Impact ADL Functions | Yes | 0.00 | 0.00 | 0.00 | binary (yes/no): impact on activities of daily life; |
| Recovery | Yes | 0.84 | 0.83 | 0.89 | binary (yes/no): the patient recovered from the relapse; |
| Severity | Yes | 0.8 | 0.79 | 0.86 | numerical (0-2): relapse severity; |
| CNS Pyramidal Tract | Yes | 0.00 | 0.00 | 0.00 | binary (yes/no): MS manifestations related to the Pyramidal tract; |
| CNS Brain Stem | Yes | 0.00 | 0.00 | 0.00 | binary (yes/no): MS manifestations related to the Brain Stem; |
| CNS Bowell & Bladder | Yes | 0.00 | 0.00 | 0.00 | binary (yes/no): MS manifestations related to Bowel and Bladder; |
| CNS Neuropsycho Functions | Yes | 0.00 | 0.00 | 0.00 | binary (yes/no): MS manifestations related to Neuropsycho functions; |
| CNS Cerebellum | Yes | 0.00 | 0.00 | 0.00 | binary (yes/no): MS manifestations related to the Cerebellum; |
| CNS Visual Functions | Yes | 0.00 | 0.00 | 0.00 | binary (yes/no): MS manifestations related to Visual Functions; |
| CNS Sensory Functions | Yes | 0.00 | 0.00 | 0.00 | binary (yes/no): MS manifestations related to Sensory Functions; |
| Hospital | Yes | 0.00 | 0.00 | 0.00 | binary (yes/no): the relapse required hospitalization; |
| Ambulatory | Yes | 0.00 | 0.00 | 0.00 | binary (yes/no): the relapse affected ambulatory capacity; |
| Corticosteroids | No | 0.00 | 0.00 | 0.00 | binary (yes/no): corticosteroids were administered; |

**Table S 2.** Database information concerning visits and relapses, which are considered dynamic information.

| Variable | Used | Missing Data Ratio (0-1) | | | Description |
|---|---|---|---|---|---|
| | | **SP Development Prediction** | **Disease Severity in the 6th Year** | **Disease Severity in the 10th Year** | |
| **MRI (Dynamic)** | | **available for 91% patients** | **available for 94% patients** | **available for 92% patients** | |
| Patient ID | No | 0.00 | 0.00 | 0.00 | - |
| Exam Date | No | 0.00 | 0.00 | 0.00 | - |
| Exam Region | No | 0.00 | 0.00 | 0.00 | the MRI region:brain, whole spinal cord, or cercival cord; |
| T1 | No | 0.00 | 0.00 | 0.00 | binary (yes/no): a T1-MRI was performed; |
| Status T1 | No | 0.80 | 0.67 | 0.29 | double binary (normal/abnormal, MS typical/atypical): T1-RMI was normal/abnormal and MS-typical/atypical; |
| Nb Lesions T1 | No | 0.94 | 0.79 | 0.35 | numerical: number of lesions found in T1-MRI; |
| T1 GdE | No | 0.00 | 0.00 | 0.00 | binary (yes/no): a T1 Gde-MRI was performed; |
| Status T1Gde | No | 0.81 | 0.67 | 0.30 | double binary (normal/abnormal, MS typical/atypical): T1 Gde-RMI was normal/abnormal and MS-typical/atypical; |
| Nb Lesions GdE | No | 0.86 | 0.72 | 0.32 | numerical: number of lesions found in T1 Gde-MRI; |
| T2 | No | 0.00 | 0.00 | 0.00 | binary (yes/no): a T2-MRI was performed; |
| Status T2 | No | 0.80 | 0.66 | 0.29 | double binary (normal/abnormal, MS typical/atypical): T2-RMI was normal/abnormal and MS-typical/atypical; |
| Nb Lesions T2 | No | 0.92 | 0.78 | 0.34 | numeric: number of lesions found in T2-MRI; |
| Nb New or Enlarging Lesions | No | 0.94 | 0.79 | 0.34 | numeric: number of new or enlarging lesions; |
| McDonald Lesions T1 Gd | No | 0.81 | 0.67 | 0.30 | numeric: number of new lesions in T1-Gde according to McDonald Criteria; |
| McDonald Lesions T2 | No | 0.77 | 0.64 | 0.28 | numeric: number of new lesions in T2 according to McDonald Criteria; |
| McDonald Lesions Infratentorial | No | 0.79 | 0.66 | 0.29 | numeric: number of new lesions in infratentorial region according to McDonald Criteria; |
| McDonald Lesions Juxtacortical | No | 0.79 | 0.67 | 0.29 | numeric: number of new lesions in juxtacortical region according to McDonald Criteria |
| McDonald Lesions Periventricular | No | 0.79 | 0.67 | 0.28 | numeric: number of new lesions in periventricular region according to McDonald Criteria; |
| Contiguous lesion extending more than 3 vertebral segments | No | 0.95 | 0.80 | 0.36 | binary (yes/no): a very atypical case in MS neuromyelitis optica; |
| **Treatments (Dynamic)** | | **available for 100% of patients** | **available for 100% of patients** | **available for 100% of patients** | |
| Patient ID | No | 0.00 | 0.00 | 0.00 | - |
| MS Specific | No | 0.00 | 0.00 | 0.00 | binary (yes/no): the drug isMS specific; |
| Medication | No | 0.00 | 0.00 | 0.00 | name of the administered drug; |
| Start Date | No | 0.00 | 0.00 | 0.00 | - |
| End Date | No | 0.00 | 0.00 | 0.00 | - |
| Dosage | No | 0.00 | 0.00 | 0.00 | - |
| Unit | No | 0.00 | 0.00 | 0.00 | - |
| Frequency | No | 0.00 | 0.00 | 0.00 | - |
| Route | No | 0.00 | 0.00 | 0.00 | - |
| Discontinuation | No | 0.00 | 0.00 | 0.00 | the specific reason for a drug discontinuation, if any; |
| **Concomitant Diseases (Dynamic)** | | **available for 27% of patients** | **available for 26% of patients** | **available for 33% of patients** | |
| Patient ID | No | 0.00 | 0.00 | 0.00 | - |
| Concomitant Disease Start Date | No | 0.00 | 0.00 | 0.00 | - |
| Concomitant Disease End Date | No | 0.00 | 0.00 | 0.00 | - |
| Concomitant Disease Name | No | 0.00 | 0.00 | 0.00 | - |

**Table S 3.** Database information concerning MRI exams, treatments, and concomitant diseases, which are considered dynamic information.

## Extracted Features for All $N$-Year Models

In Tables S4, S5, and S6 one can see the full list of extracted features. All information is ordered according to the original database order. Each $N$-year model contains the 10 static features from Table S4. With concern to the dynamic ones, its number varies, as more features are available with the passage of time. From each information (total of 36) from Table S5, it is possible to extract up to a maximum of eight features, since we applied four different mathematical operators (mean, median, standard deviation, and mode) and two segmentation windows (normal and accumulative). Thus, for each year, we can calculate up to a maximum of 288 features (36 pieces of information $\times$ 4 mathematical operators $\times$ 2 segmentation windows).

| Feature Name | Origin Tab | Feature Name | Origin Tab |
|---|---|---|---|
| Gender | Identification | Spinal Cord | Identification |
| Age of Onset | Identification | Clinical Findings | Identification |
| Supratentorial | Identification | MRI | Identification |
| Optic Pathways | Identification | Evoked Potentials | Identification |
| Brainstem Cerebellum | Identification | CSF | Identification |

**Table S 4.** The list of extracted static features.

| Feature Name | Origin Tab | Feature Name | Origin Tab |
|---|---|---|---|
| Routine | Visits | mwUpperExtrem | Visits |
| Score Pyramidal | Visits | micturitionPb | Visits |
| Score Cerebellar | Visits | fatigue | Visits |
| Cerebellar Weakness | Visits | mwLowerExtrem | Visits |
| Score BrainStem | Visits | moodPb | Visits |
| Score Sensory | Visits | EDSS | Visits |
| Score Bowel & Bladder | Visits | Impact ADL functions | Relapses |
| Score Visual | Visits | Recovery | Relapses |
| Visual Symptoms | Visits | Severity | Relapses |
| Score Mental | Visits | CNS Pyramidal Tract | Relapses |
| Score Ambulation | Visits | CNS Brain Stem | Relapses |
| gdAtaxia | Visits | CNS Bowell & Bladder | Relapses |
| dysaesthesiae | Visits | CNS Neuropsycho Functions | Relapses |
| ataxiaLowerExtrem | Visits | CNS Cerebellum | Relapses |
| paresthesiae | Visits | CNS Visual Functions | Relapses |
| cognitionPb | Visits | CNS Sensory Functions | Relapses |
| gdParesis | Visits | Hospital | Relapses |
| gdSpasticity | Visits | Ambulatory | Relapses |

**Table S 5.** The list of information from which dynamic features are extracted for each $N$-Year Model.

| $N$-Year Model | Static Features | Dynamic Features | Total Number |
|---|---|---|---|
| 1-Year Model | 10 | 36 $\times$ 4 mathematical operators $\times$ 1 segmentation window | 154 |
| 2-Year Model | 10 | Dynamic Features from 1-Year Model + 36 $\times$ 4 mathematical operators $\times$ 2 segmentation windows | 442 |
| 3-Year Model | 10 | Dynamic Features from 2-Year Model + 36 $\times$ 4 mathematical operators $\times$ 2 segmentation windows | 730 |
| 4-Year Model | 10 | Dynamic Features from 3-Year Model + 36 $\times$ 4 mathematical operators $\times$ 2 segmentation windows | 1018 |
| 5-Year Model | 10 | Dynamic Features from 4-Year Model + 36 $\times$ 4 mathematical operators $\times$ 2 segmentation windows | 1306 |

**Table S 6.** The list of extracted features for each $N$-year model.

Consequently, the number of features for each $N$-year model is presented in Table S6. Thus, each $N$-year model has 154, 442, 730, 1018 and 1306 features, respectively. Each consecutive year-model has a difference of those mentioned 288 features,

which corresponds to the number of extracted dynamic features from one year. It is important to note that the 1-Year Model has only dynamic features extracted from the normal segmentation window (144 features).

## Missing Data Imputation

In Tables S7, S8 and S9 we present the amount (ratio) of imputed values for each dynamic feature, for the SP Development, and severity at the 6$^{th}$ and 10$^{th}$ tracking years, respectively. The considered static features did not contain any missing value.

| SP Development data set | Tab | 1-Year Usual Window | 2-Year Usual Window | 2-Year Accum. Window | 3-Year Usual Window | 3-Year Accum. Window | 4-Year Usual Window | 4-Year Accum. Window | 5-year Usual Window | 5-year Accum. Window |
|---|---|---|---|---|---|---|---|---|---|---|
| Routine | Visits | 0.00 | 0.36 | 0.00 | 0.35 | 0.00 | 0.32 | 0.00 | 0.22 | 0.22 |
| Score Pyramidal | Visits | 0.34 | 0.54 | 0.32 | 0.51 | 0.28 | 0.44 | 0.25 | 0.36 | 0.18 |
| Score Cerebellar | Visits | 0.34 | 0.54 | 0.32 | 0.51 | 0.28 | 0.44 | 0.25 | 0.36 | 0.19 |
| Cerebellar Weakness | Visits | 0.00 | 0.36 | 0.00 | 0.35 | 0.00 | 0.32 | 0.00 | 0.22 | 0.00 |
| Score BrainStem | Visits | 0.34 | 0.54 | 0.32 | 0.51 | 0.29 | 0.44 | 0.25 | 0.36 | 0.19 |
| Score Sensory | Visits | 0.34 | 0.54 | 0.32 | 0.51 | 0.29 | 0.44 | 0.25 | 0.36 | 0.19 |
| Score Bowel & Bladder | Visits | 0.34 | 0.54 | 0.32 | 0.51 | 0.29 | 0.44 | 0.25 | 0.36 | 0.19 |
| Score Visual | Visits | 0.34 | 0.54 | 0.32 | 0.51 | 0.29 | 0.44 | 0.25 | 0.36 | 0.19 |
| Visual Symptoms | Visits | 0.00 | 0.36 | 0.00 | 0.35 | 0.00 | 0.32 | 0.00 | 0.22 | 0.00 |
| Score Mental | Visits | 0.34 | 0.54 | 0.32 | 0.51 | 0.29 | 0.44 | 0.25 | 0.36 | 0.19 |
| Score Ambulation | Visits | 0.34 | 0.54 | 0.32 | 0.51 | 0.29 | 0.44 | 0.25 | 0.36 | 0.19 |
| gdAtaxia | Visits | 0.00 | 0.36 | 0.00 | 0.35 | 0.29 | 0.32 | 0.00 | 0.22 | 0.00 |
| dysaesthesiae | Visits | 0.00 | 0.36 | 0.00 | 0.35 | 0.29 | 0.32 | 0.00 | 0.22 | 0.00 |
| ataxiaLowerExtrem | Visits | 0.00 | 0.36 | 0.00 | 0.35 | 0.29 | 0.32 | 0.00 | 0.22 | 0.00 |
| paresthesiae | Visits | 0.00 | 0.36 | 0.00 | 0.35 | 0.29 | 0.32 | 0.00 | 0.22 | 0.00 |
| cognitionPb | Visits | 0.00 | 0.36 | 0.00 | 0.35 | 0.29 | 0.32 | 0.00 | 0.22 | 0.00 |
| gdParesis | Visits | 0.00 | 0.36 | 0.00 | 0.35 | 0.29 | 0.32 | 0.00 | 0.22 | 0.00 |
| gdSpasticity | Visits | 0.00 | 0.36 | 0.00 | 0.35 | 0.29 | 0.32 | 0.00 | 0.22 | 0.00 |
| mwUpperExtrem | Visits | 0.00 | 0.36 | 0.00 | 0.35 | 0.29 | 0.32 | 0.00 | 0.22 | 0.00 |
| micturitionPb | Visits | 0.00 | 0.36 | 0.00 | 0.35 | 0.29 | 0.32 | 0.00 | 0.22 | 0.00 |
| fatigue | Visits | 0.00 | 0.36 | 0.00 | 0.35 | 0.29 | 0.32 | 0.00 | 0.22 | 0.00 |
| mwLowerExtrem | Visits | 0.00 | 0.36 | 0.00 | 0.35 | 0.29 | 0.32 | 0.00 | 0.22 | 0.00 |
| moodPb | Visits | 0.00 | 0.36 | 0.00 | 0.35 | 0.29 | 0.32 | 0.00 | 0.22 | 0.00 |
| EDSS | Visits | 0.00 | 0.36 | 0.00 | 0.35 | 0.29 | 0.32 | 0.00 | 0.22 | 0.00 |
| Impact ADL functions | Relapses | 0.26 | 0.67 | 0.11 | 0.72 | 0.09 | 0.78 | 0.08 | 0.72 | 0.07 |
| Recovery | Relapses | 0.81 | 0.93 | 0.72 | 0.94 | 0.68 | 0.97 | 0.67 | 0.96 | 0.66 |
| Severity | Relapses | 0.81 | 0.92 | 0.71 | 0.94 | 0.68 | 0.94 | 0.66 | 0.96 | 0.64 |
| CNS Pyramidal Tract | Relapses | 0.26 | 0.67 | 0.11 | 0.72 | 0.09 | 0.78 | 0.08 | 0.73 | 0.07 |
| CNS Brain Stem | Relapses | 0.26 | 0.67 | 0.11 | 0.72 | 0.09 | 0.78 | 0.08 | 0.73 | 0.07 |
| CNS Bowell & Bladder | Relapses | 0.26 | 0.67 | 0.11 | 0.72 | 0.09 | 0.78 | 0.08 | 0.73 | 0.07 |
| CNS Neuropsycho Functions | Relapses | 0.26 | 0.67 | 0.11 | 0.72 | 0.09 | 0.78 | 0.08 | 0.73 | 0.07 |
| CNS Cerebellum | Relapses | 0.26 | 0.67 | 0.11 | 0.72 | 0.09 | 0.78 | 0.08 | 0.73 | 0.07 |
| CNS Visual Functions | Relapses | 0.26 | 0.67 | 0.11 | 0.72 | 0.09 | 0.78 | 0.08 | 0.73 | 0.07 |
| CNS Sensory Functions | Relapses | 0.26 | 0.67 | 0.11 | 0.72 | 0.09 | 0.78 | 0.08 | 0.73 | 0.07 |
| Hospital | Relapses | 0.26 | 0.67 | 0.11 | 0.72 | 0.09 | 0.78 | 0.08 | 0.73 | 0.07 |
| Ambulatory | Relapses | 0.26 | 0.67 | 0.11 | 0.72 | 0.09 | 0.78 | 0.08 | 0.73 | 0.07 |

**Table S 7.** The ratio of imputed values, for each feature, for the SP development data set.

| | | 1-Year | 2-Year | | 3-Year | | 4-Year | | 5-year | |
|---|---|---|---|---|---|---|---|---|---|---|
| Disease Severity at 6th tracking year | Tab | Usual Window | Usual Window | Accum. Window | Usual Window | Accum. Window | Usual Window | Accum. Window | Usual Window | Accum. Window |
| Routine | Visits | 0.00 | 0.33 | 0.00 | 0.32 | 0.00 | 0.30 | 0.00 | 0.19 | 0.00 |
| Score Pyramidal | Visits | 0.35 | 0.55 | 0.33 | 0.52 | 0.30 | 0.44 | 0.25 | 0.35 | 0.18 |
| Score Cerebellar | Visits | 0.35 | 0.55 | 0.33 | 0.52 | 0.30 | 0.44 | 0.25 | 0.36 | 0.19 |
| Cerebellar Weakness | Visits | 0.00 | 0.33 | 0.00 | 0.32 | 0.00 | 0.30 | 0.00 | 0.19 | 0.00 |
| Score BrainStem | Visits | 0.35 | 0.55 | 0.33 | 0.52 | 0.30 | 0.44 | 0.25 | 0.36 | 0.19 |
| Score Sensory | Visits | 0.35 | 0.55 | 0.33 | 0.52 | 0.30 | 0.44 | 0.25 | 0.36 | 0.19 |
| Score Bowel & Bladder | Visits | 0.35 | 0.55 | 0.33 | 0.52 | 0.30 | 0.44 | 0.25 | 0.36 | 0.19 |
| Score Visual | Visits | 0.35 | 0.55 | 0.33 | 0.52 | 0.30 | 0.44 | 0.25 | 0.36 | 0.19 |
| Visual Symptoms | Visits | 0.00 | 0.33 | 0.00 | 0.32 | 0.00 | 0.30 | 0.00 | 0.19 | 0.00 |
| Score Mental | Visits | 0.35 | 0.55 | 0.33 | 0.52 | 0.30 | 0.44 | 0.25 | 0.36 | 0.19 |
| Score Ambulation | Visits | 0.35 | 0.55 | 0.33 | 0.52 | 0.30 | 0.44 | 0.25 | 0.36 | 0.18 |
| gdAtaxia | Visits | 0.00 | 0.33 | 0.00 | 0.32 | 0.00 | 0.30 | 0.00 | 0.19 | 0.00 |
| dysaesthesiae | Visits | 0.00 | 0.33 | 0.00 | 0.32 | 0.00 | 0.30 | 0.00 | 0.19 | 0.00 |
| ataxiaLowerExtrem | Visits | 0.00 | 0.33 | 0.00 | 0.32 | 0.00 | 0.30 | 0.00 | 0.19 | 0.00 |

Table 8 continued from previous page

| | | | | | | | | | | |
|---|---|---|---|---|---|---|---|---|---|---|
| paresthesiae | Visits | 0.00 | 0.33 | 0.00 | 0.32 | 0.00 | 0.30 | 0.00 | 0.19 | 0.00 |
| cognitionPb | Visits | 0.00 | 0.33 | 0.00 | 0.32 | 0.00 | 0.30 | 0.00 | 0.19 | 0.00 |
| gdParesis | Visits | 0.00 | 0.33 | 0.00 | 0.32 | 0.00 | 0.30 | 0.00 | 0.19 | 0.00 |
| gdSpasticity | Visits | 0.00 | 0.33 | 0.00 | 0.32 | 0.00 | 0.30 | 0.00 | 0.19 | 0.00 |
| mwUpperExtrem | Visits | 0.00 | 0.33 | 0.00 | 0.32 | 0.00 | 0.30 | 0.00 | 0.19 | 0.00 |
| micturitionPb | Visits | 0.00 | 0.33 | 0.00 | 0.32 | 0.00 | 0.30 | 0.00 | 0.19 | 0.00 |
| fatigue | Visits | 0.00 | 0.33 | 0.00 | 0.32 | 0.00 | 0.30 | 0.00 | 0.19 | 0.00 |
| mwLowerExtrem | Visits | 0.00 | 0.33 | 0.00 | 0.32 | 0.00 | 0.30 | 0.00 | 0.19 | 0.00 |
| moodPb | Visits | 0.00 | 0.33 | 0.00 | 0.32 | 0.00 | 0.30 | 0.00 | 0.19 | 0.00 |
| EDSS | Visits | 0.00 | 0.33 | 0.00 | 0.32 | 0.00 | 0.30 | 0.00 | 0.19 | 0.00 |
| Impact ADL functions | Relapses | 0.28 | 0.66 | 0.12 | 0.71 | 0.10 | 0.74 | 0.09 | 0.70 | 0.08 |
| Recovery | Relapses | 0.80 | 0.94 | 0.71 | 0.94 | 0.68 | 0.97 | 0.67 | 0.96 | 0.66 |
| Severity | Relapses | 0.79 | 0.92 | 0.68 | 0.94 | 0.66 | 0.94 | 0.62 | 0.96 | 0.60 |
| CNS Pyramidal Tract | Relapses | 0.28 | 0.66 | 0.12 | 0.71 | 0.10 | 0.74 | 0.09 | 0.70 | 0.08 |
| CNS Brain Stem | Relapses | 0.28 | 0.66 | 0.12 | 0.71 | 0.10 | 0.74 | 0.09 | 0.70 | 0.08 |
| CNS Bowell & Bladder | Relapses | 0.28 | 0.66 | 0.12 | 0.71 | 0.10 | 0.74 | 0.09 | 0.70 | 0.08 |
| CNS Neuropsycho Functions | Relapses | 0.28 | 0.66 | 0.12 | 0.71 | 0.10 | 0.74 | 0.09 | 0.70 | 0.08 |
| CNS Cerebellum | Relapses | 0.28 | 0.66 | 0.12 | 0.71 | 0.10 | 0.74 | 0.09 | 0.70 | 0.08 |
| CNS Visual Functions | Relapses | 0.28 | 0.66 | 0.12 | 0.71 | 0.10 | 0.74 | 0.09 | 0.70 | 0.08 |
| CNS Sensory Functions | Relapses | 0.28 | 0.66 | 0.12 | 0.71 | 0.10 | 0.74 | 0.09 | 0.70 | 0.08 |
| Hospital | Relapses | 0.28 | 0.66 | 0.12 | 0.71 | 0.10 | 0.74 | 0.09 | 0.70 | 0.08 |
| Ambulatory | Relapses | 0.28 | 0.66 | 0.12 | 0.71 | 0.10 | 0.74 | 0.09 | 0.70 | 0.08 |

**Table S 8.** The ratio of imputed values, for each feature, for the disease severity at the 6h tracking year data set.

| | | 1-Year | 2-Year | | 3-Year | | 4-Year | | 5-year | |
|---|---|---|---|---|---|---|---|---|---|---|
| **Disease Severity at 10th tracking year** | **Tab** | **Usual Window** | **Usual Window** | **Accum. Window** | **Usual Window** | **Accum. Window** | **Usual Window** | **Accum. Window** | **Usual Window** | **Accum. Window** |
| Routine | Visits | 0.00 | 0.64 | 0.00 | 0.54 | 0.00 | 0.51 | 0.00 | 0.36 | 0.00 |
| Score Pyramidal | Visits | 0.43 | 0.84 | 0.43 | 0.84 | 0.40 | 0.71 | 0.37 | 0.63 | 0.30 |
| Score Cerebellar | Visits | 0.43 | 0.84 | 0.43 | 0.84 | 0.40 | 0.71 | 0.37 | 0.64 | 0.31 |
| Cerebellar Weakness | Visits | 0.00 | 0.64 | 0.00 | 0.54 | 0.00 | 0.51 | 0.00 | 0.36 | 0.00 |
| Score BrainStem | Visits | 0.43 | 0.84 | 0.43 | 0.84 | 0.40 | 0.71 | 0.37 | 0.64 | 0.31 |
| Score Sensory | Visits | 0.43 | 0.84 | 0.43 | 0.84 | 0.40 | 0.71 | 0.37 | 0.64 | 0.31 |
| Score Bowel & Bladder | Visits | 0.43 | 0.84 | 0.43 | 0.84 | 0.40 | 0.71 | 0.37 | 0.64 | 0.31 |
| Score Visual | Visits | 0.43 | 0.84 | 0.43 | 0.84 | 0.40 | 0.71 | 0.37 | 0.64 | 0.31 |
| Visual Symptoms | Visits | 0.00 | 0.64 | 0.00 | 0.64 | 0.00 | 0.51 | 0.00 | 0.36 | 0.00 |
| Score Mental | Visits | 0.43 | 0.84 | 0.43 | 0.78 | 0.40 | 0.71 | 0.37 | 0.64 | 0.31 |
| Score Ambulation | Visits | 0.43 | 0.84 | 0.43 | 0.78 | 0.40 | 0.71 | 0.37 | 0.64 | 0.30 |
| gdAtaxia | Visits | 0.00 | 0.64 | 0.00 | 0.54 | 0.00 | 0.51 | 0.00 | 0.36 | 0.00 |
| dysaesthesiae | Visits | 0.00 | 0.64 | 0.00 | 0.54 | 0.00 | 0.51 | 0.00 | 0.36 | 0.00 |
| ataxiaLowerExtrem | Visits | 0.00 | 0.64 | 0.00 | 0.54 | 0.00 | 0.51 | 0.00 | 0.36 | 0.00 |
| paresthesiae | Visits | 0.00 | 0.64 | 0.00 | 0.54 | 0.00 | 0.51 | 0.00 | 0.36 | 0.00 |
| cognitionPb | Visits | 0.00 | 0.64 | 0.00 | 0.54 | 0.00 | 0.51 | 0.00 | 0.36 | 0.00 |
| gdParesis | Visits | 0.00 | 0.64 | 0.00 | 0.54 | 0.00 | 0.51 | 0.00 | 0.36 | 0.00 |
| gdSpasticity | Visits | 0.00 | 0.64 | 0.00 | 0.54 | 0.00 | 0.51 | 0.00 | 0.36 | 0.00 |
| mwUpperExtrem | Visits | 0.00 | 0.64 | 0.00 | 0.54 | 0.00 | 0.51 | 0.00 | 0.36 | 0.00 |
| micturitionPb | Visits | 0.00 | 0.64 | 0.00 | 0.54 | 0.00 | 0.51 | 0.00 | 0.36 | 0.00 |
| fatigue | Visits | 0.00 | 0.64 | 0.00 | 0.54 | 0.00 | 0.51 | 0.00 | 0.36 | 0.00 |
| mwLowerExtrem | Visits | 0.00 | 0.64 | 0.00 | 0.54 | 0.00 | 0.51 | 0.00 | 0.36 | 0.00 |
| moodPb | Visits | 0.00 | 0.64 | 0.00 | 0.54 | 0.00 | 0.51 | 0.00 | 0.36 | 0.00 |
| EDSS | Visits | 0.00 | 0.64 | 0.00 | 0.54 | 0.00 | 0.51 | 0.00 | 0.36 | 0.00 |
| Impact ADL functions | Relapses | 0.07 | 0.72 | 0.07 | 0.63 | 0.06 | 0.69 | 0.04 | 0.60 | 0.03 |
| Recovery | Relapses | 0.39 | 0.97 | 0.80 | 0.97 | 0.81 | 0.98 | 0.81 | 0.94 | 0.79 |
| Severity | Relapses | 0.30 | 0.97 | 0.82 | 0.97 | 0.82 | 0.97 | 0.81 | 0.94 | 0.78 |
| CNS Pyramidal Tract | Relapses | 0.07 | 0.72 | 0.07 | 0.63 | 0.06 | 0.69 | 0.04 | 0.60 | 0.03 |
| CNS Brain Stem | Relapses | 0.07 | 0.72 | 0.07 | 0.63 | 0.06 | 0.69 | 0.04 | 0.60 | 0.03 |
| CNS Bowell & Bladder | Relapses | 0.07 | 0.72 | 0.07 | 0.63 | 0.06 | 0.69 | 0.04 | 0.60 | 0.03 |
| CNS Neuropsycho Functions | Relapses | 0.07 | 0.72 | 0.07 | 0.63 | 0.06 | 0.69 | 0.04 | 0.60 | 0.03 |
| CNS Cerebellum | Relapses | 0.07 | 0.72 | 0.07 | 0.63 | 0.06 | 0.69 | 0.04 | 0.60 | 0.03 |
| CNS Visual Functions | Relapses | 0.07 | 0.72 | 0.07 | 0.63 | 0.06 | 0.69 | 0.04 | 0.60 | 0.03 |
| CNS Sensory Functions | Relapses | 0.07 | 0.72 | 0.07 | 0.63 | 0.06 | 0.69 | 0.04 | 0.60 | 0.03 |
| Hospital | Relapses | 0.07 | 0.72 | 0.07 | 0.63 | 0.06 | 0.69 | 0.04 | 0.60 | 0.03 |
| Ambulatory | Relapses | 0.07 | 0.72 | 0.07 | 0.63 | 0.06 | 0.69 | 0.04 | 0.60 | 0.03 |

**Table S 9.** The ratio of imputed values, for each feature, for the disease severity at the 10h tracking year data set.

## Feature Selection - Filter Methods

In Tables S10, S11, and S12, we present the 50 set of selected features using two filter methods: the correlation coefficient and AUC. Due to the heterogeneity in terms of mathematical operations, segmentation (accumulative and normal), and due to clinical interpretability issues, we only show its clinical source, along with its mean absolute values. Clinical sources are ordered alphabetically. Clinical sources with less than 10 appearances were discarded, as they were considered non-significant (appearance below 0.10).

| Clinical Source | 1-Year Model<br>Appearance (0-1)<br>Correlation Coeff<br>AUC | 2-Year Model<br>Appearance (0-1)<br>Correlation Coeff<br>AUC | 3-Year Model<br>Appearance (0-1)<br>Correlation Coeff<br>AUC | 4-Year Model<br>Appearance (0-1)<br>Correlation Coeff<br>AUC | 5-Year Model<br>Appearance (0-1)<br>Correlation Coeff<br>AUC |
|---|---|---|---|---|---|
| Age of Onset | 1.00<br>0.40±0.06<br>0.73±0.04 | 0.95<br>0.40±0.06<br>0.73±0.04 | 1.00<br>0.40±0.06<br>0.73±0.04 | 1.00<br>0.40±0.06<br>0.73±0.04 | 1.00<br>0.40±0.06<br>0.73±0.04 |
| Ambulatory | 0.85<br>0.16±0.01<br>0.54±0.03 | 0.86<br>0.28±0.04<br>0.46±0.03 | 0.69<br>0.28±0.03<br>0.44±0.02 | 0.25<br>0.31±0.03<br>0.42±0.02 | |
| BrainStem Cerebellum | 0.99<br>0.31±0.06<br>0.65±0.03 | 1.00<br>0.31±0.06<br>0.65±0.03 | 1.00<br>0.31±0.06<br>0.65±0.03 | 0.85<br>0.31±0.06<br>0.65±0.03 | 0.47<br>0.36±0.05<br>0.67±0.02 |
| CNS Bowell & Bladder | 0.85<br>0.16±0.01<br>0.54±0.03 | 0.04<br>0.17±0.00<br>0.52±0.00 | 0.80<br>0.29±0.02<br>0.66±0.03 | 0.63<br>0.30±0.01<br>0.67±0.03 | 0.30<br>0.31±0.01<br>0.69±0.03 |
| CNS BrainStem | 1.00<br>0.11±0.07<br>0.57±0.04 | 0.81<br>0.23±0.02<br>0.48±0.04 | 0.60<br>0.26±0.01<br>0.46±0.04 | 0.12<br>0.27±0.01<br>0.49±0.11 | |
| CNS Cerebellum | 1.00<br>0.14±0.07<br>0.58±0.04 | 0.75<br>0.23±0.03<br>0.60±0.06 | 0.20<br>0.28±0.03<br>0.63±0.03 | 0.12<br>0.30±0.02<br>0.62±0.07 | |
| CNS Neuropsycho Functions | 0.89<br>0.21±0.01<br>0.62±0.03 | 0.99<br>0.25±0.04<br>0.70±0.07 | 0.90<br>0.28±0.02<br>0.71±0.08 | 0.74<br>0.29±0.01<br>0.76±0.03 | 0.32<br>0.30±0.02<br>0.78±0.03 |
| CNS Pyramidal Tract | 0.37<br>0.09±0.06<br>0.53±0.02 | 0.18<br>0.24±0.05<br>0.48±0.07 | 0.91<br>0.36±0.07<br>0.70±0.05 | 0.99<br>0.35±0.06<br>0.70±0.05 | 0.37<br>0.37±0.05<br>0.70±0.05 |
| CNS Sensory Functions | 0.99<br>0.10±0.07<br>0.57±0.04 | 0.97<br>0.24±0.03<br>0.50±0.07 | 0.75<br>0.27±0.02<br>0.45±0.02 | 0.22<br>0.30±0.02<br>0.430±0.02 | |
| CNS Visual Functions | 0.93<br>0.06±0.05<br>0.53±0.03 | 0.31<br>0.22±0.03<br>0.55±0.07 | 0.35<br>0.27±0.01<br>0.43±0.02 | | |
| EDSS | 1.00<br>0.26±0.10<br>0.69±0.06 | 1.00<br>0.30±0.07<br>0.71±0.05 | 1.00<br>0.33±0.07<br>0.73±0.05 | 1.00<br>0.37±0.07<br>0.73±0.06 | 1.00<br>0.41±0.07<br>0.75±0.05 |
| Hospital | 0.60<br>0.05±0.05<br>0.51±0.01 | 0.49<br>0.24±0.05<br>0.60±0.07 | 0.21<br>0.29±0.02<br>0.63±0.05 | 0.18<br>0.32±0.05<br>0.66±0.02 | |
| Impact ADL Functions | | | 0.12<br>0.29±0.00<br>0.44±0.02 | | |
| MRI | 0.98<br>0.07±0.07<br>0.52±0.02 | 0.03<br>0.20±0.00<br>0.57±0.00 | | | |
| mwLowerExtrem | | | 0.55<br>0.26±0.01<br>0.43±0.00 | | |
| Routine | | 0.46<br>0.27±0.01<br>0.43±0.00 | 0.65<br>0.27±0.01<br>0.43±0.00 | 0.16<br>0.30±0.03<br>0.42±0.02 | |
| Score Ambulation | | 0.56<br>0.22±0.02<br>0.44±0.03 | | 0.12<br>0.30±0.03<br>0.58±0.10 | 0.21<br>0.33±0.03<br>0.67±0.01 |
| Score Bowel & Bladder | 0.42<br>0.10±0.04<br>0.52±0.02 | 1.00<br>0.26±0.04<br>0.56±0.05 | 0.50<br>0.29±0.02<br>0.59±0.02 | 0.27<br>0.31±0.02<br>0.61±0.03 | |
| Score BrainStem | 0.96<br>0.26±0.05<br>0.57±0.03 | 1.00<br>0.32±0.06<br>0.63±0.05 | 1.00<br>0.34±0.05<br>0.64±0.05 | 1.00<br>0.37±0.06<br>0.67±0.05 | 1.00<br>0.40±0.06<br>0.70±0.04 |
| Score Cerebellar | 0.27<br>0.04±0.04<br>0.52±0.02 | 0.88<br>0.26±0.04<br>0.58±0.04 | 0.98<br>0.32±0.05<br>0.62±0.04 | 0.99<br>0.35±0.05<br>0.66±0.05 | 1.00<br>0.39±0.06<br>0.71±0.04 |

**Table 10 continued from previous page**

| | | | | | |
|---|---|---|---|---|---|
| Score Mental | 0.37<br>0.19±0.03<br>0.53±0.02 | 1.00<br>0.26±0.04<br>0.58±0.04 | 0.73<br>0.28±0.02<br>0.60±0.03 | 0.72<br>0.30±0.02<br>0.62±0.03 | 0.91<br>0.39±0.05<br>0.67±0.04 |
| Score Pyramidal | 0.12<br>0.07±0.05<br>0.51±0.02 | 0.78<br>0.25±0.03<br>0.57±0.05 | 0.43<br>0.28±0.02<br>0.59±0.04 | 0.26<br>0.31±0.03<br>0.62±0.05 | |
| Score Sensory | 0.97<br>0.22±0.06<br>0.64±0.05 | 0.96<br>0.24±0.05<br>0.63±0.05 | 0.64<br>0.30±0.04<br>0.65±0.05 | 0.53<br>0.24±0.05<br>0.63±0.05 | 0.21<br>0.33±0.03<br>0.69±0.15 |
| Score Visual | | 0.43<br>0.25±0.04<br>0.44±0.04 | 0.12<br>0.26±0.00<br>0.43±0.00 | | |

**Table S 10.** The selected features with correlation coefficient and AUC, for predicitng SP development.

| Clinical Source | 1-Year Model<br>Appearance (0-1)<br>Correlation Coeff<br>AUC | 2-Year Model<br>Appearance (0-1)<br>Correlation Coeff<br>AUC | 3-Year Model<br>Appearance (0-1)<br>Correlation Coeff<br>AUC | 4-Year Model<br>Appearance (0-1)<br>Correlation Coeff<br>AUC | 5-Year Model<br>Appearance (0-1)<br>Correlation Coeff<br>AUC |
|---|---|---|---|---|---|
| Age of Onset | 0.97<br>0.14±0.06<br>0.57±0.04 | | | 0.97<br>0.14±0.0<br>60.57±0.04 | |
| Ambulatory | 0.86<br>0.08±0.04<br>0.54±0.01 | | | | |
| ataxiaLowerExtrem | 0.10<br>0.14±0.00<br>0.52±0.00 | | | | |
| BrainStem Cerebellum | 0.55<br>0.07±0.04<br>0.53±0.01 | | | | |
| CNS Bowell & Bladder | 0.91<br>0.10±0.04<br>0.54±0.02 | | | | |
| CNS BrainStem | 0.99<br>0.18±0.05<br>0.57±0.02 | | | | |
| CNS Cerebellum | 1.00<br>0.21±0.07<br>0.59±0.03 | 0.79<br>0.29±0.04<br>0.63±0.01 | | | |
| CNS Pyramidal Tract | 0.30<br>0.05±0.02<br>0.53±0.01 | | 0.16<br>0.34±0.03<br>0.67±0.02 | | |
| CNS Sensory Functions | 0.35<br>0.07±0.06<br>0.54±0.02 | | | | |
| CNS Visual Functions | 0.43<br>0.07±0.03<br>0.54±0.01 | | | | |
| CSF | 0.40<br>0.07±0.03<br>0.54±0.01 | | | | |
| Clinical Findings | 0.97<br>0.16±0.04<br>0.55±0.01 | | | | |
| EDSS | 1.00<br>0.44±0.07<br>0.76±0.05 | 1.00<br>0.47±0.04<br>0.77±0.04 | 1.00<br>0.49±0.05<br>0.78±0.04 | 1.00<br>0.52±0.06<br>0.80±0.04 | 1.00<br>0.54±0.06<br>0.82±0.04 |
| Evoked Potentials | 0.93<br>0.12±0.04<br>0.55±0.02 | | 0.21<br>0.29±0.02<br>0.63±0.05 | | |
| Gender | 0.99<br>0.22±0.05<br>0.60±0.02 | | | | |
| gdSpasticity | 0.63<br>0.15±0.02<br>0.52±0.01 | | | | |

**Table 11 continued from previous page**

| | | | | | |
|---|---|---|---|---|---|
| Impact ADL Functions | 0.13<br>0.10±0.06<br>0.53±0.02 | | | | |
| MRI | 0.40<br>0.11±0.05<br>0.53±0.01 | | | | |
| Hospital | | 0.70<br>0.24±0.01<br>0.65±0.02 | | | |
| Recovery | 0.99<br>0.18±0.10<br>0.58±0.04 | 0.42<br>0.26±0.03<br>0.64±0.02 | | | |
| Routine | 0.06<br>0.12±0.07<br>0.53±0.02 | | | | |
| Score Ambulation | 1.00<br>0.19±0.04<br>0.61±0.04 | 1.00<br>0.27±0.04<br>0.65±0.02 | 1.00<br>0.32±0.02<br>0.69±0.02 | 1.00<br>0.36±0.03<br>0.72±0.02 | 1.00<br>0.44±0.04<br>0.75±0.02 |
| Score Bowel & Bladder | 1.00<br>0.20±0.03<br>0.58±0.03 | 1.00<br>0.33±0.04<br>0.66±0.02 | 1.00<br>0.38±0.03<br>0.71±0.03 | 0.83<br>0.39±0.03<br>0.73±0.02 | 0.83<br>0.40±0.02<br>0.74±0.02 |
| Score BrainStem | 1.00<br>0.27±0.07<br>0.65±0.03 | 0.96<br>0.28±0.04<br>0.65±0.02 | 0.29<br>0.33±0.03<br>0.68±0.02 | | |
| Score Cerebellar | 1.00<br>0.40±0.05<br>0.67±0.03 | 1.00<br>0.38±0.04<br>0.68±0.03 | 0.99<br>0.38±0.04<br>0.69±0.02 | 1.00<br>0.40±0.04<br>0.72±0.02 | 0.79<br>0.43±0.03<br>0.74±0.02 |
| Score Mental | 1.00<br>0.28±0.06<br>0.63±0.04 | 0.96<br>0.31±0.02<br>0.65±0.02 | 0.96<br>0.33±0.02<br>0.68±0.02 | 0.51<br>0.34±0.02<br>0.71±0.02 | 0.10<br>0.38±0.04<br>0.73±0.01 |
| Score Pyramidal | 1.00<br>0.36±0.10<br>0.69±0.07 | 1.00<br>0.42±0.04<br>0.72±0.03 | 1.00<br>0.42±0.04<br>0.71±0.03 | 1.00<br>0.46±0.05<br>0.74±0.03 | 1.00<br>0.48±0.04<br>0.76±0.03 |
| Score Sensory | 1.00<br>0.24±0.07<br>0.61±0.04 | 0.81<br>0.28±0.03<br>0.64±0.02 | 0.64<br>0.30±0.04<br>0.65±0.05 | | |
| Score Visual | 0.93<br>0.09±0.04<br>0.55±0.02 | 0.47<br>0.28±0.02<br>0.63±0.01 | 0.47<br>0.31±0.03<br>0.67±0.02 | | |
| Score Severity | 0.99<br>0.15±0.04<br>0.54±0.02 | | | | |
| Spinal Cord | 0.26<br>0.07±0.03<br>0.53±0.01 | | | | |
| Supratentorial | 0.39<br>0.08±0.04<br>0.53±0.01 | | | | |

**Table S 11.** The selected features with correlation coefficient and AUC, for predicitng disease severity at the 6th year of tracking.

| **Clinical Source** | **1-Year Model**<br>**Appearance (0-1)**<br>**Correlation Coeff**<br>**AUC** | **2-Year Model**<br>**Appearance (0-1)**<br>**Correlation Coeff**<br>**AUC** | **3-Year Model**<br>**Appearance (0-1)**<br>**Correlation Coeff**<br>**AUC** | **4-Year Model**<br>**Appearance (0-1)**<br>**Correlation Coeff**<br>**AUC** | **5-Year Model**<br>**Appearance (0-1)**<br>**Correlation Coeff**<br>**AUC** |
|---|---|---|---|---|---|
| Age of Onset | 1.00<br>0.29±0.05<br>0.64±0.03 | 0.91<br>0.30±0.04<br>0.64±0.03 | 0.34<br>0.32±0.04<br>0.65±0.02 | 0.34<br>0.34±0.03<br>0.67±0.02 | 0.22<br>0.34±0.02<br>0.67±0.02 |
| Ambulatory | 0.86<br>0.08±0.04<br>0.54±0.01 | 1.00<br>0.17±0.00<br>0.59±0.02 | | | |
| BrainStem Cerebellum | 0.73<br>0.07±0.04<br>0.53±0.02 | | | | |
| CNS Bowell & Bladder | 0.97<br>0.12±0.04<br>0.54±0.02 | 0.37<br>0.17±0.00<br>0.60±0.01 | | | |

**Table 12 continued from previous page**

| | | | | | |
|---|---|---|---|---|---|
| CNS BrainStem | 0.94<br>0.07±0.06<br>0.54±0.02 | | | | |
| CNS Cerebellum | 1.00<br>0.11±0.06<br>0.57±0.03 | 0.71<br>0.19±0.03<br>0.62±0.02 | | | |
| CNS Neuropsycho Functions | | 0.45<br>0.18±0.01<br>0.60±0.02 | | | |
| CNS Pyramidal Tract | 0.98<br>0.08±0.07<br>0.55±0.03 | 0.48<br>0.19±0.03<br>0.60±0.02 | 0.55<br>0.28±0.04<br>0.65±0.02 | 0.55<br>0.31±0.04<br>0.66±0.02 | 0.32<br>0.33±0.04<br>0.67±0.01 |
| CNS Sensory Functions | 0.67<br>0.04±0.04<br>0.53±0.02 | 0.39<br>0.20±0.03<br>0.61±0.02 | | | |
| CNS Visual Functions | 0.87<br>0.06±0.04<br>0.54±0.02 | | | | |
| CSF | 0.77<br>0.08±0.04<br>0.54±0.02 | | | | |
| Clinical Findings | 0.97<br>0.16±0.04<br>0.55±0.01 | | | | |
| EDSS | 1.00<br>0.46±0.03<br>0.75±0.03 | 1.00<br>0.42±0.07<br>0.74±0.05 | 1.00<br>0.44±0.07<br>0.74±0.06 | 1.00<br>0.46±0.07<br>0.76±0.05 | 1.00<br>0.48±0.07<br>0.77±0.04 |
| Evoked Potentials | 0.67<br>0.07±0.04<br>0.53±0.02 | | | | |
| Gender | 0.28<br>0.04±0.04<br>0.52±0.02 | | | | |
| Hospital | 0.74<br>0.05±0.04<br>0.54±0.02 | 1.00<br>0.30±0.06<br>0.65±0.02 | 0.97<br>0.32±0.04<br>0.66±0.02 | 0.97<br>0.33±0.04<br>0.67±0.02 | 0.78<br>0.34±0.04<br>0.68±0.02 |
| Impact ADL Functions | 0.95<br>0.04±0.05<br>0.53±0.02 | 0.35<br>0.17±0.00<br>0.60±0.01 | | | |
| Score Ambulation | 1.00<br>0.22±0.02<br>0.65±0.02 | 1.00<br>0.20±0.03<br>0.64±0.03 | 0.95<br>0.25±0.03<br>0.66±0.02 | 0.95<br>0.29±0.03<br>0.67±0.02 | 0.70<br>0.31±0.02<br>0.68±0.02 |
| Score Bowel & Bladder | 1.00<br>0.21±0.04<br>0.62±0.03 | 1.00<br>0.26±0.04<br>0.63±0.03 | 1.00<br>0.30±0.04<br>0.66±0.03 | 1.00<br>0.32±0.03<br>0.68±0.02 | 0.98<br>0.34±0.03<br>0.69±0.02 |
| Score BrainStem | 0.96<br>0.13±0.05<br>0.56±0.02 | 0.87<br>0.23±0.03<br>0.62±0.02 | 0.12<br>0.25±0.02<br>0.64±0.01 | 0.12<br>0.28±0.01<br>0.65±0.01 | |
| Score Cerebellar | 1.00<br>0.19±0.05<br>0.61±0.03 | 1.00<br>0.23±0.04<br>0.63±0.02 | 1.00<br>0.27±0.04<br>0.65±0.02 | 1.00<br>0.33±0.05<br>0.70±0.04 | 1.00<br>0.35±0.05<br>0.71±0.03 |
| Score Mental | 1.00<br>0.31±0.03<br>0.68±0.03 | 1.00<br>0.30±0.04<br>0.66±0.03 | 0.87<br>0.29±0.03<br>0.66±0.03 | 0.87<br>0.31±0.02<br>0.68±0.02 | 0.94<br>0.34±0.03<br>0.68±0.02 |
| Score Pyramidal | 0.99<br>0.22±0.05<br>0.58±0.03 | 1.00<br>0.26±0.03<br>0.62±0.02 | 1.00<br>0.30±0.04<br>0.65±0.02 | 1.00<br>0.35±0.04<br>0.67±0.02 | 1.00<br>0.40±0.06<br>0.70±0.03 |
| Score Sensory | 0.10<br>0.04±0.02<br>0.51±0.01 | 0.82<br>0.25±0.02<br>0.62±0.02 | 0.11<br>0.25±0.02<br>0.63±0.02 | 0.11<br>0.28±0.01<br>0.65±0.01 | |
| Score Visual | 0.90<br>0.09±0.05<br>0.55±0.02 | 0.83<br>0.24±0.03<br>0.62±0.02 | 0.26<br>0.25±0.02<br>0.63±0.01 | 0.26<br>0.28±0.02<br>0.66±0.02 | |
| Score Severity | 0.988<br>0.14±0.04<br>0.53±0.01 | | | | |

**Table 12 continued from previous page**

| | |
|---|---|
| Supratentorial | 0.91<br>0.11±0.04<br>0.54±0.02 |

**Table S 12.** The selected features with correlation coefficient and AUC, for predicitng disease severity at the 10th year of tracking.
